# The effects of oral clefts on hospital use throughout the lifespan

**DOI:** 10.1186/1472-6963-12-58

**Published:** 2012-03-09

**Authors:** George L Wehby, Dorthe Almind Pedersen, Jeffrey C Murray, Kaare Christensen

**Affiliations:** 1 Department of Health Management and Policy, College of Public Health, University of Iowa, 105 River Street, N248 CPHB, Iowa City, IA 52242, USA; 2 Department of Epidemiology, University of Southern Denmark, Odense, Denmark; 3 Department of Pediatrics, College of Medicine, University of Iowa, Iowa City, IA, USA

## Abstract

**Background:**

Oral clefts are one of the most common birth defects worldwide. They require multiple healthcare interventions and add significant burden on the health and quality of life of affected individuals. However, not much is known about the long term effects of oral clefts on health and healthcare use of affected individuals. In this study, we evaluate the effects of oral clefts on hospital use throughout the lifespan.

**Methods:**

We estimate two-part regression models for hospital admission and length of stay for several age groups up to 68 years of age. The study employs unique secondary population-based data from several administrative inpatient, civil registration, demographic and labor market databases for 7,670 individuals born with oral clefts between 1936 and 2002 in Denmark, and 220,113 individuals without oral clefts from a 5% random sample of the total birth population from 1936 to 2002.

**Results:**

Oral clefts significantly increase hospital use for most ages below 60 years by up to 233% for children ages 0-10 years and 16% for middle age adults. The more severe cleft forms (cleft lip with palate) have significantly larger effects on hospitalizations than less severe forms.

**Conclusions:**

The results suggest that individuals with oral clefts have higher hospitalization risks than the general population throughout most of the lifespan.

## Background

Birth defects are common health problems with life-long implications. For example, about 3% of all children in the United States (US) are born with birth defects [1]. Oral clefts or cleft lip and/or cleft palate are one of the most prevalent birth defects and include clefts of the lip with or without the palate or clefts of the palate only. More than 6,500 affected babies were born with oral clefts in 2001 in the US [1]. Oral cleft incidence ranges between 1 per 500 to 1 per 2500 births and varies by ancestral origin and socioeconomic status [2]. The majority of cases occur without other major birth defects [3,4]. A complex etiology of genetic and environmental factors likely contributes to oral clefts [5-11].

Oral clefts are associated with difficulties in feeding, growth, cognitive development, speech and behavior and require several surgical, medical, nutritional, dental, and other healthcare interventions [12,13]. Oral clefts may significantly increase the risk of neonatal and infant mortality, especially when present with other birth defects [5,14-18]. Furthermore, oral clefts may increase long-term all-cause mortality and suicide risks [19].

Several studies have found reductions in the quality of life and psychosocial performance among affected individuals that is partly related to low satisfaction with facial appearance [18,20-25]. The effects of oral clefts may also extend through adulthood and reduce psychosocial, educational, and economic achievement [26-30].

Identifying the effects of oral clefts on long-term healthcare use is particularly important for assessing the healthcare needs of affected individuals throughout life and devising healthcare practices and policies that address these needs. Oral clefts significantly increase individual healthcare expenditures during childhood by up to 8 times [31]. However, studies of the long-term effects of oral clefts on healthcare use based on large population-based samples are extremely rare. One study reported that adults with oral clefts have increased risks of psychiatric hospitalizations due to mental retardation and substance abuse (RR = 1.8) and autism (RR = 5.9) [32]. Additionally, that study found that adults with cleft palate alone have a higher admission risk due to autism. To our knowledge, no other studies have evaluated the long-term hospital use of affected individuals. Such an evaluation is crucial especially with previous reports of increased mental health-related hospitalizations and long-term risks of specific cancer types, even though there is no significant overall cancer increase risk [33].

In this paper, we assess the effects of oral clefts on hospital use from birth through 68 years of age using an extensive and unique population-level dataset from Denmark. One inherent limitation in conducting such studies has been the lack of appropriate data sources and health registries that allow following affected individuals throughout life. The Danish national population-level healthcare, demographic, and economic datasets provide an important resource and methodological strength for these studies. Denmark and other Scandinavian countries have the highest prevalence rates of oral clefts among populations of Caucasian ancestry (about 1 in 500 births) [34]. Another advantage of employing data from Denmark is that individuals face essentially the same health insurance availability, which may be a strong confounder for studies of the effects of birth defects on healthcare use in settings where insurance availability varies between individuals with birth defects and unaffected individuals. Health insurance is a strong predictor of healthcare use, and individuals with birth defects may face added difficulties in obtaining insurance in countries with multiple payers and insurance systems. Direct adjustment for insurance status may not be adequate due to self-selection into insurance status in such countries based on unobserved factors (adverse self-selection).

## Methods

### Data source and study sample

The study uses linked data from various national and population-level registries and datasets in Denmark. These datasets provide individual-level data on several outcomes and variables, have been used in several studies, and are known to be of high quality with low missing data rates [35]. Statistics Denmark administers the access to these datasets and ensures that security, confidentiality, and anonymity are maintained while allowing micro-level data analysis. The study investigators accessed the assembled study datasets at Statistics Denmark via a secure virtual private network (VPN) connection. The datasets provided by Statistics Denmark have no individual or organizational identifiers and are stored on servers located within Statistics Denmark. The study was approved by the by the Danish Data Protection Agency (Case No. 92/229 MC) and the University of Iowa IRB (Protocol # 200708740).

The study datasets include the Danish Facial Cleft Database, the Danish National Patient Registry, the Danish Civil Registration System, the Danish Demographic Database, and the Integrated Database for Labor Market Research. Almost all births (about 99%) with oral clefts since 1936 in Denmark have been registered in the Danish Facial Cleft Database [34]. The registry is assembled from various data sources including surgical records from the two hospitals where all oral cleft repairs surgeries are done - cleft surgeries have been centralized in Denmark since the mid 1930s. The registry also uses the records of the National Institute for Defects of Speech to which midwives and other health professionals are required to report observed cases with oral clefts. The registry includes data on presence of other malformations or syndromes. The majority of registered cases (including most of the cases born in the 1960s or later) can be linked at Statistics Denmark to the other national registries through the unique personal identification numbers.

The Danish National Patient Registry is maintained by the Danish National Board of Health and includes data from local health authorities in order to facilitate planning in the health care system. The dataset provides data on somatic hospitalizations including admission/discharge dates, diagnoses (using standard codes ICD 8 and 10), and operations. The Danish Civil Registration System includes information about marital and vital status and residence reported through local municipalities. The Danish Demographic Database is constructed by Statistics Denmark from several public administrative databases and includes data on cause of death, date and country of migration, and relationship to others sharing the same dwelling. The socioeconomic data are obtained from the Integrated Database for Labor Market Research which is based on a number of registers [36].

The cases in the study sample include 7,670 individuals born between 1936 and 2002 with oral clefts but without other major birth defects such as neural tube defects or recognized syndromes as identified from the Danish Facial Cleft Database [37]. About 9.6% of individuals in the Danish Facial Cleft Database have another major malformation besides oral clefts or a genetic syndrome [38]. As mentioned above, this sample includes virtually all individuals born with oral clefts and without other major birth defects during this period in Denmark. The controls in the study sample include 220,113 individuals without oral clefts from a randomly selected sample of about 5% of the total population of births in Denmark each year from 1936 to 2002. Both cases and controls are limited to individuals born in Denmark who were alive on or born after January 1st, 1981 (the beginning date for hospitalization data availability), lived (at least for some time) in Denmark between 1981 and 2004, and have complete information on the study variables.

### Empirical model and statistical analysis

We employ a panel data design where individuals have a measurement of hospitalizations for each year that they are observed in the sample. We model hospitalizations in a given year as a function of cleft status and other variables that may affect hospital use. Specifically, we use the following function:

(1) $$HOSPITALIZATIONS_{it}=\alpha_{0}+\beta CLEFT_{i}+\overset{D}{\underset{d=1}{\sum }}\lambda_{d}DEMOGRAPHIC_{dit}+\overset{Y}{\underset{y=1}{\sum }}\varsigma_{y}YEAR_{yit}+v_{it}$$

where for individual i, the number of days hospitalized in year t (*HOSPITALIZATIONS*) are a function of whether the individual was born with a cleft or not (*CLEFT)*, demographic characteristics including the individual's age, sex, and number of days spent in Denmark (*DEMOGRAPHIC*), and year fixed effects (*YEAR*). Hospitalization risks are expected to vary over age. Cleft repairs are usually completed early in life (within the first two years of life). Some follow-up surgeries may be performed during adolescence. Since cleft repair surgeries in Denmark are centralized and surgical repair costs are covered by the universal health insurance program, there is limited variation in age at repair. After adolescence, there are generally no increased hospitalizations due to cleft repair, and any additional hospitalization risks are expected to be almost entirely due to physical and mental health co-morbidities of oral clefts. In order to capture changes in hospitalization risks due to oral clefts over age, we model hospitalizations during several age groups that are feasible with the available data including 0-9, 10-19, 20-29, 30-39, 40-49, 50-59 and 60-68 years, which cover most of the life span. We do not exclude hospitalizations due to cleft repair surgeries during childhood and adolescence in order to capture the "total" effects of oral clefts on hospitalizations, and not only effects through cleft co-morbidities.

Given that parental socioeconomic and demographic factors may affect the child's cleft risks and hospitalization outcomes, we also adjust in (Equation 1) models for age groups 0-9 and 10-19 years for maternal and paternal education, employment and income in year t-1 (*PARENT_SOCIOECONOMIC*), maternal and paternal ages in year t (*PARENT_DEMOGRAPHIC)*, maternal marital status in year t-1 (*PARENT_MARITAL)*, and area-level characteristics in year t-1 including maternal county of residence and county's population density (*AREA*) as follows:

(2) $$\begin{array}{ccc} HOSPITALIZATIONS & =\;\alpha_{0}^{\prime }+\beta \prime CLEFT_{i}+\overset{S}{\underset{s=1}{\sum }}\delta_{s}PARENT\text{\_}SOCIOECONOMIC_{si\left(t-1\right)}+\overset{D}{\underset{d=1}{\sum }}\lambda_{d}^{\prime }DEMOGRAPHIC_{dit} & \\ & \;\;\;\;\;\;+\overset{P}{\underset{p=1}{\sum }}\kappa_{p}PARENT\text{\_}DEMOGRAPHIC_{pit}+\overset{R}{\underset{r=1}{\sum }}\pi_{r}PARENT\text{\_}MARITAL_{ri\left(t-1\right)}\\ & \;\;\;\;\;\;+\overset{A}{\underset{a=1}{\sum }}{\gamma_{a}AREA}_{ai\left(t-1\right)}+\overset{Y}{\underset{y=1}{\sum }}\varsigma_{y}^{\prime }YEAR_{yit}+u_{it} \end{array}$$

We use (') to indicate that the coefficients vary between (Equation 1) and (Equation 2). We include the socioeconomic, marital status and area variables at time t-1 given that child hospitalizations may have reverse effects on parental income, employment, marital status, and residential location.

We do not include parental socioeconomic and demographic characteristics for ages older than 19 years as these are not available for a large proportion of these age groups (links between parent and children are available beginning for the 1953 birth cohort and are nearly complete from 1960) [39]. Furthermore, we do not include in the main model for age groups older than 19 individual-level socioeconomic characteristics because, as mentioned above, cleft status may have negative impacts on the individual's educational attainment, wealth, employment, and marriage status, which in turn may affect hospitalizations. Therefore, estimating the "total" effects of cleft status on hospitalizations requires omitting these variables from the model. However, we also evaluate the "direct" effects of oral clefts on hospitalizations separately from the indirect effects on psychosocial and economic performance by estimating an additional specification of (Equation 1) for ages 20 years and older that controls for individual-level education, income, marital status, employment, and marital status (*SOCIOECONOMIC*) as well as county of residence and county's population density (*AREA*) given that oral cleft status may affect the individual's social mobility and residential location. All these additional socioeconomic and area controls are measured at year t-1 in order to account for the potential reverse effects of hospitalizations on these variables.

(3) $$\begin{array}{ccc} HOSPITALIZATIONS_{it} & ={\alpha^{\prime \prime }}_{0}+\beta^{\prime \prime }CLEFT_{i}+\overset{S}{\underset{s=1}{\sum }}{\delta^{\prime \prime }}_{s}SOCIOECONOMIC_{si\left(t-1\right)}+\overset{D}{\underset{d=1}{\sum }}{\lambda^{\prime \prime }}_{d}DEMOGRAPHIC_{dit} & \\ & +\overset{A}{\underset{a=1}{\sum }}{\gamma^{\prime \prime }}_{a}AREA_{ai\left(t-1\right)}+\overset{Y}{\underset{y=1}{\sum }}{\varsigma^{\prime \prime }}_{y}YEAR_{yit}+e_{it} \end{array}$$

We use (") to indicate that the coefficients are different from the above two equations. In addition to estimating the effects of any cleft, we estimate the effects of cleft types (cleft lip alone, cleft palate alone, and cleft lip with palate) on hospitalizations given that oral cleft status effects may vary by cleft type/severity. Tables 1 and 2 list the distribution of all model variables for ages 19 years or younger and older than 19 years, respectively.

**Table 1:** Sample Characteristics - Age group 0-9 and 10-19 years

Variables	Age group			
	
	0-9 years		10-19 years	
	
	controls	cases	controls	cases
Total number of observations	665,588	26,178	683,315	27,500

Total number of individuals	90,791	3,597	98,163	3,889

Male	342,157 (51.41%)	15,684 (59.91%)	351,112 (51.38%)	16,756 (60.93%)

Age (years)	4.54 (2.86)	4.59 (2.85)	14.54 (2.88)	14.53 (2.87)

Hospitalized at least once	57,287 (8.61%)	6,949 (26.55%)	38,312 (5.61%)	4,475 (16.27%)

Days hospitalized (among those with at least one hospitalization)	5.23 (11.34)	8.08 (11.11)	4.67 (9.60)	5.64 (6.33)

Maternal age (years)	32.80 (5.38)	32.74 (5.51)	41.39 (5.42)	41.27 (5.51)

Paternal age (years)	35.54 (6.17)	35.59 (6.27)	44.20 (6.11)	44.17(6.27)

Maternal income (DKK)	146,463 (90,890)	142,991 (84,593)	149,570 (101,353)	148,123 (113,759)

Paternal income (DKK)	242,329 (189,762)	233,934 (171,085)	259,505 (224,802)	248,957 (205,933)

Maternal education				

Primary and lower secondary	221,224 (33.24%)	9,928 (37.92%)	277,152 (40.56%)	12,247 (44.53%)

Upper and post-secondary	267,481 (40.19%)	9,957 (38.04%)	250,418 (36.65%)	9,656 (35.11%)

Tertiary	176,883 (26.58%)	6,293 (24.04%)	155,745 (22.79%)	5,597 (20.35%)

Paternal education				

Primary and lower secondary	175,444 (26.36%)	7,808 (29.83%)	210,665 (30.83%)	9,604 (34.92%)

Upper and post-secondary	323,376 (48.59%)	12,503 (47.76%)	317,229 (46.43%)	12,307 (44.75%)

Tertiary	166,768 (25.06%)	5,867 (22.41%)	155,421 (22.75%)	5,589 (20.32%)

Maternal occupational status				

Self-employed	26,960 (4.05%)	943 (3.60%)	50,088 (7.33%)	1,873 (6.81%)

Employed	480,752 (72.23%)	18,666 (71.30%)	509,599 (74.58%)	20,000 (72.73%)

Unemployed/others	157,876 (23.72%)	6,569 (25.09%)	123,628 (18.09%)	5,627 (20.46%)

Paternal occupational status				

Self-employed	65,939 (9.91%)	2,715 (10.37%)	97,360 (14.25%)	3,955 (14.38%)

Employed	531,849 (79.91%)	20,552 (78.51%)	519,868 (76.08%)	20,328 (73.92%)

Unemployed/others	67,800 (10.19%)	2,911 (11.12%)	66,087 (9.67%)	3,217 (11.70%)

Maternal marital status				

Married	449,196 (67.49%)	17,113 (65.37%)	531,119 (77.73%)	20,912 (76.04%)

Cohabiting	139,977 (21.03%)	5,700 (21.77%)	54,594 (7.99%)	2,213 (8.05%)

Single	76,415 (11.48%)	3,365 (12.85%)	97,602 (14.28%)	4,375 (15.91%)

Maternal urbanization				

> = 1000 Inh/km2	107,350 (16.13%)	3,877 (14.81%)	90,742 (13.28%)	3,948 (14.36%)

500-999 Inh/km2	103,838 (15.60%)	4,237 (16.19%)	103,881 (15.20%)	4,227 (15.37%)

200-499 Inh/km2	126,009 (18.93%)	4,840 (18.49%)	130,914 (19.16%)	4,817 (17.52%)

100-199 Inh/km2	96,566 (14.51%)	3,651 (13.95%)	104,857 (15.35%)	4,323 (15.72%)

50-99 Inh/km2	143,713 (21.59%)	5,719 (21.85%)	156,472 (22.90%)	6,058 (22.03%)

< 50 Inh/km2	88,112 (13.24%)	3,854 (14.72%)	96,449 (14.11%)	4,127 (15.01%)

Exposure time (days living in Denmark per year)	347.50 (62.19)	348.12 (61.87)	364.67 (10.52)	364.83 (9.58)

Note: The table reports the distribution of the model variables in the case and control groups for age groups 0-19 years. For continuous variables, the mean is reported with the standard deviation in parenthesis. For categorical variables, the frequency is reported with the percentage in parenthesis. "Inh" indicates inhabitants.

**Table 2:** Sample Characteristics - Age group 20-29, 30-39, 40-49, 50-59 and 60-68 years

Variables	Age group									
	
	20-29 years		30-39 years		40-49 years		50-59 years		60-68 years	
	
	controls	cases	controls	Cases	controls	cases	controls	cases	controls	cases
Number of observations	809,974	30,665	843,807	28,199	776,786	22,259	461,190	11,978	114,708	2,594

Male	414,779 (51.21%)	18,839 (61.43%)	430,615 (51.03%)	17,207 (61.02%)	394,148 (50.74%)	13,088 (58.80%)	231,696 (50.24%)	6,902 (57.62%)	56,652 (49.39%)	1,489 (57.41%)

Age (years)	24.56 (2.87)	24.49 (2.86)	34.53 (2.87)	34.42 (2.87)	44.29 (2.84)	44.17 (2.84)	53.84 (2.79)	53.79 (2.78)	62.52 (2.17)	62.35 (2.09)

Exposure time (days)	363.56 (19.43)	363.65 (18.96)	364.24 (15.50)	364.29 (15.35)	364.40 (14.31)	364.25 (15.72)	363.94 (17.97)	363.78 (18.44)	363.01 (23.85)	362.03 (29.29)

Hospitalized at least once	94,494 (11.67%)	4,299 (14.02%)	93,509 (11.08%)	3,331 (11.81%)	65,961 (8.49%)	2,194 (9.86%)	45,083 (9.78%)	1,327 (11.08%)	14,784 (12.89%)	346 (13.34%)

Days hospitalized (if hosp at least once)	5.52 (9.99)	5.98 (9.61)	5.83 (10.94)	6.42 (12.38)	7.79 (14.44)	8.42 (15.89)	9.41 (17.18)	9.66 (18.07)	10.86 (18.57)	10.68 (17.73)

Income (DKK)	123,945 (77,726)	121,800 (75,051)	192,523 (121,500)	189,219 (116,759)	217,822 (178,648)	212,897 (173,192)	239,396 (224,678)	228,357 (160,773)	212,304 (196,313)	206,992 (146,882)

Education										

Primary and lower secondary	297,153 (36.69%)	13,766 (44.89%)	245,626 (29.11%)	9,867 (34.99%)	267,417 (34.43%)	8,666 (38.93%)	175,494 (38.05%)	5,148 (42.98%)	52,074 (45.40%)	1,226 (47.26%)

Upper and post-secondary	435,834 (53.81%)	14,422 (47.03%)	383,077 (45.40%)	12,027 (42.65%)	324,811 (41.81%)	8,762 (39.36%)	187,931 (40.75%)	4,423 (36.93%)	42,703 (37.23%)	931 (35.89%)

Tertiary	76,987 (9.50%)	2,477 (8.08%)	215,104 (25.49%)	6,305 (22.36%)	184,558 (23.76%)	4,831 (21.70%)	97,765 (21.20%)	2,407 (20.10%)	19,931 (17.38%)	437 (16.85%)

Occupational status										

Self-employed	13,765 (1.70%)	463 (1.51%)	51,060 (6.05%)	1,422 (5.04%)	73,477 (9.46%)	1,834 (8.24%)	45,158 (9.79%)	1,052 (8.78%)	8,433 (7.35%)	140 (5.40%)

Employed	611,023 (75.44%)	22,030 (71.84%)	668,622 (79.24%)	21,350 (75.71%)	593,681 (76.43%)	16,070 (72.20%)	316,158 (68.55%)	7,673 (64.06%)	37,438 (32.64%)	888 (34.23%)

Unemployed/others	185,186 (22.86%)	8,172 (26.65%)	124,125 (14.71%)	5,427 (19.25%)	109,628 (14.11%)	4,355 (19.57%)	99,874 (21.66%)	3,253 (27.16%)	68,837 (60.01%)	1,566 (60.37%)

Marital status										

Married	107,168 (13.23%)	2,949 (9.62%)	457,235 (54.19%)	12,211 (43.30%)	526,766 (67.81%)	12,636 (56.77%)	324,544 (70.37%)	7,214 (60.23%)	79,749 (69.52%)	1,544 (59.52%)

Cohabiting	233,266 (28.80%)	7,158 (23.34%)	169,010 (20.03%)	5,090 (18.05%)	72,799 (9.37%)	2,131 (9.57%)	30,029 (6.51%)	822 (6.86%)	5,773 (5.03%)	133 (5.13%)

Single	469,540 (57.97%)	20,558 (67.04%)	217,562 (25.78%)	10,898 (38.65%)	177,221 (22.81%)	7,492 (33.66%)	106,617 (23.12%)	3,942 (32.91%)	29,186 (25.44%)	917 (35.35%)

Urbanization										

> = 1000 Inh/km2	181,837 (22.45%)	6,499 (21.19%)	161,546 (19.14%)	5,553 (19.69%)	127,076 (16.36%)	3,967 (17.82%)	72,867 (15.80%)	2,161 (18.04%)	17,711 (15.44%)	444 (17.12%)

500-999 Inh/km2	144,758 (17.87%)	5,371 (17.52%)	135,991 (16.12%)	4,387 (15.56%)	124,759 (16.06%)	3,478 (15.63%)	74,472 (16.15%)	1,911 (15.95%)	18,459 (16.09%)	438 (16.89%)

200-499 Inh/km2	154,331 (19.05%)	5,740 (18.72%)	158,589 (18.79%)	5,175 (18.35%)	151,396 (19.49%)	4,121 (18.51%)	93,885 (20.36%)	2,271 (18.96%)	23,636 (20.61%)	494 (19.04%)

100-199 Inh/km2	108,477 (13.39%)	4,273 (13.93%)	121,933 (14.45%)	4,092 (14.51%)	117,405 (15.11%)	3,270 (14.69%)	69,050 (14.97%)	1,729 (14.43%)	16,727 (14.58%)	348 (13.42%)

50-99 Inh/km2	139,364 (17.21%)	5,539 (18.06%)	169,834 (20.13%)	5,779 (20.49%)	164,398 (21.16%)	5,003 (22.48%)	96,798 (20.99%)	2,628 (21.94%)	24,287 (21.17%)	607 (23.40%)

< 50 Inh/km2	81,207 (10.03%)	3,243 (10.58%)	95,914 (11.37%)	3,213 (11.39%)	91,752 (11.81%)	2,420 (10.87%)	54,118 (11.73%)	1,278 (10.67%)	13,888 (12.11%)	263 (10.14%)

Exposure time	363.56	363.65	364.24	364.29	364.40	364.25	363.94	363.78	363.01	362.03

(days living in Denmark per year)	(19.43)	(18.96)	(15.50)	(15.35)	(14.31)	(15.72)	(17.97)	(18.44)	(23.85)	(29.29)

The number of days hospitalized per year includes a high proportion of zero values (zero-inflated measure) and is skewed to the right due to the small proportion of lengthy hospitalizations. Two-part models [40], commonly referred to as Hurdle models in the case of count dependent variables, are typically used for outcomes with such a distribution [41,42]. The first part estimates the probability of hospital admission, and the second part estimates the function of hospitalization days for those who were hospitalized. In addition to accommodating the zero-inflated and right-skewed dependent variable, the two-part model evaluates if oral clefts have different effects on hospitalization propensity and on length of stay after admission. For example, individuals with clefts may have a higher propensity of being admitted to the hospital due to potentially facing higher risks for certain chronic conditions (such as mental health issues or cancer). However, once admitted, they may or may not have a different length of stay.

We use a two-part model with logistic regression to estimate the probability function for hospital admission and zero-truncated Poisson regression to fit the function of length of stay for admitted individuals. We evaluate the effects of oral clefts in both functions and estimate the overall combined incremental effect on hospitalization days from both models. We estimate the standard error of the overall incremental effect using bootstrap with 500 replications. In order to account for the multiple yearly observations of the same individual over the entire age group when evaluating the oral cleft effects on hospitalization probability and length of stay for those hospitalized, we estimate the variance-covariance matrix for the regression coefficients using a robust Huber-type estimator [43]. This estimator accounts for the repeated observations of the same individual over time in the used panel data design. Given that the logistic regression and zero-truncated Poisson regression models are expected to provide consistent estimates of the regression coefficients, adjusting the standard error estimates is appropriate in this case for accounting for repeated measurements.

## Results

### Sample description

**Figure 1:**
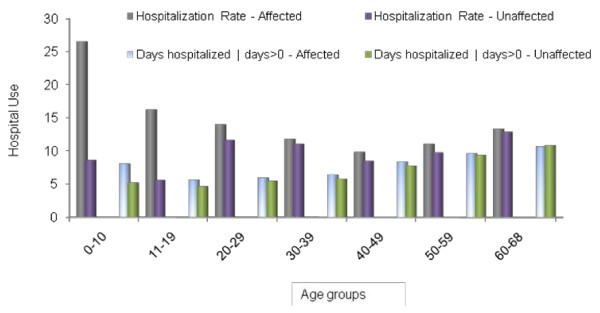
**Unadjusted Hospitalization Rates and Days**. The graph shows the unadjusted hospital admission rates and length of stay among those hospitalized for individuals with and without oral clefts.

Figure 1 shows the hospital admission rates and length of stay by age for the cleft and control groups. Individuals with oral clefts have higher hospital admission rates than individuals without clefts at all age groups but the differences decrease significantly with age. Also, hospitalized individuals with clefts have on average a longer length of stay for most age groups except at 60-68 years of age, with the differences significantly decreasing with age. The average annual hospital admission rates among the individuals with oral clefts range from 27% during ages 0-9 years to 13% during ages 60-68 years. The lowest hospital admission rate for affected individuals is 10% for ages 40-49 years. The average annual hospital admission rate for individuals without clefts ranges from 9% for ages 0-9 years to 13% for ages 60-68, with a lowest admission rate of 6% for ages 11-19 years.

The changes in hospital admission rates by age vary between the cleft and control groups. Among individuals with oral clefts, admission rates continuously decrease with age until age 40-49 years and increase thereafter. Among individuals without clefts, admission rates increase markedly from ages 11-19 to 20-29 years by about two times, decrease slightly after that until age 40-49 years, and increase thereafter. Unlike differences between individuals with and without clefts in admission rate changes over age, there is no difference between the two groups in the direction of changes in hospital length of stay over age.

### Effects of oral clefts on hospitalizations

**Figure 2:**
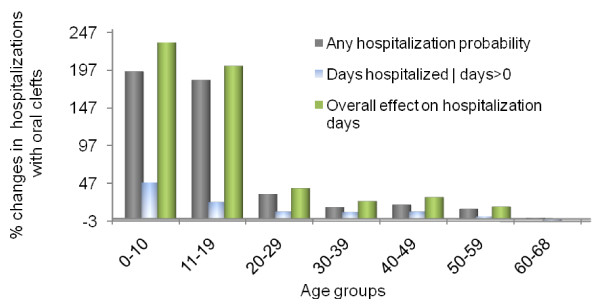
**Adjusted Effects of Oral Clefts on Hospitalizations**. The graph shows the effects of oral clefts on the probability of hospital admission and on the length of stay when admitted for individuals with oral clefts relative to individuals without clefts. Also shown are the "total" effects on hospitalization days that combine both of the aforementioned effects. All effects are estimated from the two-part regression models and are adjusted for all the covariates listed in (Equation 1) for ages 20 years and older and for covariates listed in (Equation 2) for younger ages.

**Figure 3:**
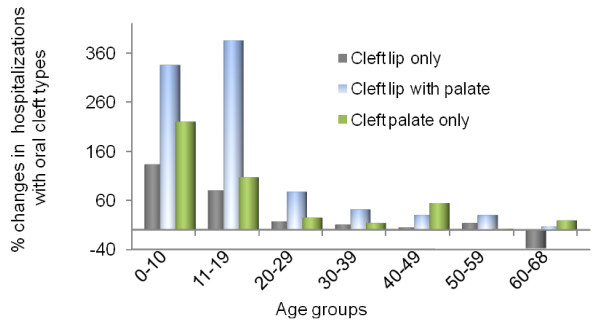
**Total Adjusted Effects of Cleft Types on Hospitalization**. The graph shows the "total" effects of oral cleft types on hospitalizations days for individuals with oral clefts relative to individuals without clefts. These effects combine the separate effects of cleft types on the probability of hospital admission and on the length of stay when admitted and are estimated from the two-part regression models. The effects are adjusted for all the covariates listed in (Equation 1) for ages 20 years and older and for covariates listed in (Equation 2) for younger ages.

Table 3 reports the effects of oral cleft status and cleft type on the probability of hospital admission, hospitalization days among those admitted, and the total combined effects on hospitalization days from the two-part model for each of the study age groups. Additional file 1: Tables S1, Additional file 2: Table S2, Additional file 3: Table S3, Additional file 4: Table S4, Additional file 5: Table S5, Additional file 6: Table S6, Additional file 7: Table S7, report detailed regression results. Also reported in Table 3 is the percentage change in hospitalizations with oral clefts relative to the control group without clefts, which are also depicted in Figures 2 and 3 for the overall cleft status and cleft type effects, respectively. Oral clefts significantly increase hospitalizations in most age groups below 60 years, with generally decreasing effects by age. Oral clefts have insignificant effects on hospitalizations between 60 and 68 years of age.

**Table 3:** Incremental Effects of Cleft Status and Type on Hospitalizations by Age Group

Age group/Cleft Measure	Hospitalization Probability	Hospitalization Days|Days > 0	Total Effect on Hospitalization Days
Age 0-9 years			

Cleft Status	0.168****	2.5****	1.05****

	(0.003)	(0.17)	(0.02)

	[195.1%]	[47.8%]	[233.2%]

	{188.0%,202.1%}	{41.6%,54.0%}	{226.3%,241.3%}

Cleft lip	0.099****	1.2****	0.6****

	(0.004)	(0.2)	(0.03)

	[115.0%]	[22.9%]	[133.2%]

	{106.0%,124.1%}	{15.4%,30.4%}	{122.0%,143.9%}

Cleft lip with palate	0.245****	3.37****	1.51****

	(0.005)	(0.27)	(0.028)

	[284.5%]	[64.4%]	[335.3%]

	{273.5%,296.2%}	{54.3%,74.6%}	{324.0%,348.5%}

Cleft palate	0.158****	2.38****	0.99****

	(0.006)	(0.31)	(0.03)

	[183.5%]	[45.5%]	[219.9%]

	{170.3%,197.3%}	{34.0%,56.9%}	{206.3%,234.7%}

Age 10-19 years			

Cleft Status	0.103****	1.03****	0.53****

	(0.003)	(0.12)	(.01)

	[183.6%]	[22.1%]	[202.3%]

	{174.0%,193.7%}	{16.8%,27.2%}	{193.3%,212.6%}

Cleft lip	0.043****	0.26	0.21****

	(0.004)	(0.21)	(0.02)

	[76.6%]	[5.6%]	[80.2%]

	{63.1%,89.5%}	{-3.1%,14.3%}	{66.9%,94.3%}

Cleft lip with palate	0.203****	1.43****	1.01****

	(0.005)	(0.15)	(0.02)

	[361.9%]	[30.6%]	[385.5%]

	{344.9%,378.4%}	{24.4%,36.9%}	{368.9%,403.5%}

Cleft palate	0.054****	0.63**	0.28****

	(0.004)	(0.28)	(0.02)

	[96.3%]	[13.5%]	[106.9%]

	{81.1%,111.9%}	{1.6%,25.4%}	{93.9%,123.0%}

Age 20-29 years			

Cleft Status	0.038****	0.53***	0.26****

	(0.003)	(0.2)	(0.02)

	[32.6%]	[9.6%]	[40.4%]

	{27.8%,37.1%}	{2.6%,16.7%}	{34.3%,46.1%}

Cleft lip	0.014***	0.35	0.11***

	(0.004)	(0.54)	(0.04)

	[12.0%]	[6.3%]	[17.1%]

	{4.2%,19.0%}	{-13.1%,25.8%}	{4.9%,28.8%}

Cleft lip with palate	0.084****	0.43**	0.5****

	(0.005)	(0.17)	(0.03)

	[72.0%]	[7.8%]	[77.6%]

	{63.2%,80.6%}	{1.7%,14.0%}	{68.7%,85.8%}

Cleft palate	0.015***	0.84**	0.16****

	(0.004)	(0.39)	(0.04)

	[12.9%]	[15.2%]	[24.8%]

	{5.2%,19.7%}	{1.4%,28.9%}	{14.0%,36.6%}

Age 30-39 years			

Cleft Status	0.017****	0.51*	0.15****

	(0.003)	(0.27)	(0.02)

	[15.3%]	[8.7%]	[23.2%]

	{10.8%,19.9%}	{-0.5%,17.9%}	{15.7%,29.9%}

Cleft lip	0.008*	0.21	0.07*

	(0.004)	(0.41)	(0.04)

	[7.2%]	[3.6%]	[10.8%]

	{-0.5%,14.5%}	{-10.1%,17.4%}	{-0.6%,20.7%}

Cleft lip with palate	0.038****	0.56	0.27****

	(0.005)	(0.44)	(0.04)

	[34.3%]	[9.6%]	[41.8%]

	{26.4%,43.0%}	{-5.0%,24.3%}	{31.1%,54.0%}

Cleft palate	0.003	0.72	0.09*

	(0.004)	(0.54)	(0.05)

	[2.7%]	[12.3%]	[13.%]

	{-4.7%,10.1%}	{-6.0%,30.6%}	{-2.0%,29.4%}

Age 40-49 years			

Cleft Status	0.016****	0.75*	0.19****

	(0.003)	(0.42)	(0.03)

	[18.8%]	[9.6%]	[28.7%]

	{12.6%,25.9%}	{-1.0%,20.3%}	{18.5%,38.1%}

Cleft lip	-0.002	0.58	0.03

	(0.004)	(0.71)	(0.06)

	[-2.4%]	[7.4%]	[4.5%]

	{-12.6%,8.0%}	{-10.3%,25.3%}	{-12.7%,23.0%}

Cleft lip with palate	0.03****	-0.36	0.2****

	(0.005)	(0.49)	(0.04)

	[35.3%]	[-4.6%]	[30.2%]

	{23.3%,46.5%}	{-16.9%,7.7%}	{16.6%,42.6%}

Cleft palate	0.019****	2.51**	0.36****

	(0.005)	(1.02)	(0.07)

	[22.4%]	[32.2]	[54.4%]

	{10.4%,34.6%}	{1.9%,57.9%}	{33.0%,74.6%}

Age 50-59 years			

Cleft Status	0.013***	0.31	0.15***

	(0.004)	(0.57)	(0.05)

	[13.3%]	[3.3%]	[16.3%]

	{5.3%,21.9%}	{-8.6%,15.2%}	{5.1%,28.2%}

Cleft lip	-0.001	1.43	0.13

	(0.007)	(1.32)	(0.12)

	[-1.0%]	[15.2%]	[14.1%]

	{-14.5%,12.1%}	{-12.2%,42.5%}	{-12.2%,39.7%}

Cleft lip with palate	0.025****	0.48	0.28***

	(0.007)	(0.81)	(0.08)

	[25.6%]	[5.1%]	[30.4%]

	{11.5%,40.1%}	{-11.7%,21.8}	{12.5%,48.1%}

Cleft palate	0.013*	-1.06	0.02

	(0.007)	(0.84)	(0.09)

	[13.3%]	[-11.3%]	[2.2%]

	{-1.2%,28.0%}	{-28.8%,6.2%}	{-17.8%,21.8%}

Age 60-68 years			

Cleft Status	0.002	-0.3	-0.01

	(0.009)	(1.06)	(0.14)

	[1.6%]	[-2.8%]	[-0.7%]

	{-12.0%,15.7%}	{-21.9%,16.5%}	{-20.8%,19.1%}

Cleft lip	-0.022	-2.25	-0.52**

	(0.015)	(1.38)	(0.22)

	[-17.1%]	[-20.7%]	[-37.1%]

	{-39.3%,5.4%}	{-45.6%,4.2%}	{-67.8%,-6.2%}

Cleft lip with palate	0.011	-0.13	0.1

	(0.014)	(1.51)	(0.23)

	[8.5%]	[-1.2%]	[7.1%]

	{-12.2%,29.6%}	{-28.5%,26.0%}	{-24.3%,39.0%}

Cleft palate	0.013	1.04	0.27

	(0.019)	(2.38)	(0.27)

	[10.1%]	[9.6%]	[19.3%]

	{-18.3%,38.9%}	{-33.3%,52.4%}	{-18.9%,57.8%}

Note: Standard errors of effects are in parentheses; the % changes in hospitalizations relative to the unaffected control group due to the cleft effects are listed in brackets (the 95% confidence intervals of these % changes are included in curly brackets); the effects are estimated following (Equation 1) for age groups 20-29 and older and (Equation 2) for younger age groups; * = p < 1; ** = p < 0.05; *** = p < 0.01; **** = p < 0.001. The effects are adjusted for all the covariates listed in (Equation 1) for ages 20 years and older and for covariates listed in (Equation 2) for younger ages.

Oral clefts increase the probability of hospital admission in all age groups below 60 years and increase the length of stay among those hospitalized for age groups below 50 years. The increase in admission probability ranges from 0.17 for age group 0-10 years to 0.013 for age group 50-59 years. This is equivalent to a 195% to 13% increase in hospital admission probability relative to the control individuals without oral clefts. The effects on hospitalization days per year among those admitted range from 2.5 days for age group 0-10 years to 0.8 days for age group 40-49 years, which represent 48% to 10% increase in length of stay relative to controls. The total effect of oral clefts on hospitalization days from the two-part model (which combines effects on probability of admission and hospitalization days conditional on use) ranges from 1 day per year for age group 0-10 years to 0.16 days per year for age group 50-59 years. These represent 233% to 16% increases in hospitalization days relative to the controls. The total absolute oral cleft effect in the 0-10 age group is twice as large as that in the 11-19 age group and four times as large as that in the 20-29 age group. However, the percentage increase in hospitalization days relative to the controls is still very high in the 11-19 age group at 202%.

Among the cleft types, cleft lip with palate has the largest total effects on hospitalizations for all age groups below 60 except for age group 40-49 years for which cleft palate alone has the largest total effect. Cleft lip alone has the smallest effect compared to the other cleft types. Cleft lip with palate significantly increases hospital admission probability per year by increments ranging from 0.25 for the 0-10 age group to 0.024 for age 50-59 years. These represent 285% to 26% increases in admission probability, respectively, relative to the controls. The largest increase in hospitalization probability with cleft lip with palate relative to the controls is 362% for the 11-19 age group. Cleft lip with palate significantly increases length of stay among those hospitalized only up to age 29 years, with the increases ranging between 3.4 days per year for the 0-10 age group to 0.4 days per year for the 20-29 age group, which represent 64% to 8% increases in hospital length of stay relative to controls, respectively. The total effects of cleft lip with palate on hospitalization days range from 1.5 days per year for the 0-10 age group to 0.3 days per year for the 50-59 age group, which represent 335% to 30% increase relative to controls, respectively. The largest increase in total hospitalizations with cleft lip with palate relative to controls is 386% for the 11-19 age group.

In contrast, cleft palate alone significantly increases length of stay among those hospitalized for all age groups below 50 years except for the 30-39. The largest effects are for the 0-10 and 40-49 age groups, for which length of stay is increased by more than 2 days per year (about 46% and 32% increase relative to controls, respectively). The total effects of cleft palate alone on hospitalization days range from 1 day per year for the 0-10 age group to 0.3 days per year for ages 40-49 years, which exceed the effects for the intermediate age groups and represent hospitalization increases of 219% and 54% relative to the controls, respectively.

Cleft lip alone generally has no significant effects on hospitalizations beyond 29 years of age (only a very small and marginally significant effect for the 30-39 age group). The total effects of cleft lip alone on increasing hospitalizations range from 0.6 days per year for the 0-10 age group to 0.1 day per year for the 20-29 year old group, which are equivalent to 133% to 17% increase relative to the controls, respectively. Cleft lip alone decreases hospitalization days for ages 60-68 years by half a day per year (37% decrease relative to the controls).

### Effects on hospitalizations through socioeconomic status

Table 4 reports the effects of oral clefts on hospital admission and length of stay among those hospitalized for age groups older than 19 years adjusting for individual-level socioeconomic and area characteristics (Equation 3). Additional file 3: Table S3, Additional file 4: Table S4, Additional file 5: Table S5, Additional file 6: Table S6 and Additional file 7: Table S7 report detailed regression results. The goal is to evaluate the extent to which the total cleft effects on hospitalizations described above are mediated by these characteristics which may also be affected by oral clefts. Adjusting for individual-level socioeconomic and area characteristics significantly decreases the effects of oral clefts on hospital admission and/or length of stay among those hospitalized beginning at age group 30-39 years, with larger decreases in the oral cleft effects over age. The oral cleft effects on length of stay decrease by more than half and become insignificant for all age groups above 29 years. Further, the cleft effects on hospital admission decrease by 50% for ages above 39 years and become insignificant for age group 50-59 years.

**Table 4:** Incremental Effects of Cleft Status on Hospitalizations by Age Group Adjusting for Own Socioeconomic Characteristics

Age group/Cleft Measure	Hospitalization Probability	Hospitalization Days|Days > 0
Age 20-29 years	0.036****	0.44**

	(0.003)	(0.19)

	[30.8%]	[8.0%]

	{26.5%,35.3%}	{1.0%,14.9%}

Age 30-39 years	0.015****	0.22

	(0.003)	(0.25)

	[13.5%]	[3.8%]

	{4.0%,15.8%}	{-5.2%,14.0%}

Age 40-49 years	0.008***	0.34

	(0.003)	(0.38)

	[9.4%]	[4.4%]

	{4.0%,15.8%}	{-5.2%,14.0%}

Age 50-59 years	0.006	-0.09

	(0.004)	(0.52)

	[6.1%]	[-1.0%]

	{-1.3%,13.6%}	{-11.8%,9.9%}

Age 60-68 years	-0.003	-0.77

	(0.009)	(0.98)

	[-2.3%]	[-7.1%]

	{-15.0%,11.1%}	{-24.7%,10.5%}

Note: Standard errors of effects are in parentheses; the % changes in hospitalizations relative to the unaffected control group due to the cleft effects are listed in brackets (the 95% confidence intervals of these % changes are included in curly brackets); The effects are estimated from (Equation 3), adjusting for the covariates in that model; * = p < 1; ** = p < 0.05; *** = p < 0.01; **** = p < 0.001.

The larger declines in the oral cleft effects by age after adjusting for individual-level socioeconomic and area characteristics and the overall minimal effect of this adjustment for the 20-29 age group strongly suggest that these declines are due to the indirect cleft effects on hospitalization through affecting individual-level psychosocial and economic performance rather than due to reflecting the effects of parental-level socioeconomic status that are unobserved for the older age groups. Improved individual-level socioeconomic performance has the expected negative effects on hospitalizations, but is negatively correlated with oral cleft status (see Tables 2 and Additional file 3: Table S3, Additional file 4: Table S4, Additional file 5: Table S5, Additional file 6: Table S6 and Additional file 7: Table S7).

In order to further check whether including the individual-level socioeconomic and area characteristics for the older age groups is reflecting their unobserved family socioeconomic background characteristics, we re-estimate the models for ages 19 years and younger excluding all parental socioeconomic, demographic, and area characteristics (Equation 1). Table 5 reports the oral cleft effects on hospitalizations for age groups 0-9 and 10-19 years after this exclusion (detailed regression results are in Additional file 1: Table S1 and Additional file 2: Table S2). The effects from this model are virtually the same as those in the full model adjusting for parental socioeconomic, demographic, and area variables (in Table 3). This provides assurance that the models for the older age groups that do not adjust for parental and family background characteristics are unlikely to be biased by such unobserved family backgrounds and that the changes in cleft effects on hospitalizations with adjusting for individual-level socioeconomic and area characteristics is due to the cleft effects on these characteristics.

**Table 5:** Incremental Effects of Cleft Status on Hospitalizations by Age Group Excluding Parental Socioeconomic and Demographic Characteristics

Age group/Cleft Measure	Hospitalization Probability	Hospitalization Days|Days > 0	Total Effect on Hospitalization Days
Age 0-9 years			

Cleft Status	0.171****	2.55****	1.08****
	(0.003)	(0.17)	(0.018)
	[198.6%]	[48.8%]	[240%]

	{191.7%,205.8%}	{42.6%,55.1%}	{233.1%,248.4%}

Cleft lip	0.099****	1.207****	0.60****
	(0.004)	(0.200)	(0.02)
	[115.0%]	[23.1%]	[133.3%]

	{105.7%,124.0%}	{15.6%,30.6%}	{123.6%,144.9%}

Cleft lip with palate	0.248****	3.420****	1.55****
	(0.005)	(0.271)	(0.03)
	[288.0%]	[65.4%]	[344.4%]

	{276.7%,299.1%}	{55.3%,75.5%}	{330.4%,356.4%}

Cleft palate	0.166****	2.488****	1.05****
	(0.006)	(0.316)	(0.03)
	[192.8%]	[47.6%]	[233.3%]

	{178.5%,206.3%}	{35.7%,59.4%}	{219.3%,247.0%}

Age 10-19 years			

Cleft Status	0.107****	1.05****	0.55****
	(0.003)	(0.12)	(0.01)
	[190.7%]	[22.5%]	[210.0%]

	{181.0%,201.1%}	{17.2%,27.6%}	{202.3%,221.3%}

Cleft lip	0.045****	0.261	0.22****
	(0.004)	(0.210)	(0.02)
	[80.2%]	[5.6%]	[84.0%]

	{66.0%,92.9%}	{-3.2%,14.4%}	{69.9%,98.5%}

Cleft lip with palate	0.208****	1.445****	1.04****
	(0.005)	(0.144)	(0.02)
	[370.8%]	[30.9%]	[396.9%]

	{354.5%,387.5%}	{24.9%,37.0%}	{379.9%,416.5%}

Cleft palate	0.058****	0.675**	0.31****
	(0.005)	(0.289)	(0.02)
	[103.4%]	[14.5%]	[118.3%]

	{87.8%,120.0%}	{2.3%,26.6%}	{101.4%,133.4%}

Age group/Cleft Measure	Hospitalization Probability	Hospitalization Days|Days > 0	Total Effect on Hospitalization Days

Age 0-9 years			

Cleft Status	0.171****	2.55****	1.08****
	(0.003)	(0.17)	(0.018)
	[198.6%]	[48.8%]	[240%]

	{191.7%,205.8%}	{42.6%,55.1%}	{233.1%,248.4%}

Cleft lip	0.099****	1.207****	0.60****
	(0.004)	(0.200)	(0.02)
	[115.0%]	[23.1%]	[133.3%]

	{105.7%,124.0%}	{15.6%,30.6%}	{123.6%,144.9%}

Cleft lip with palate	0.248****	3.420****	1.55****
	(0.005)	(0.271)	(0.03)
	[288.0%]	[65.4%]	[344.4%]

	{276.7%,299.1%}	{55.3%,75.5%}	{330.4%,356.4%}

Cleft palate	0.166****	2.488****	1.05****
	(0.006)	(0.316)	(0.03)
	[192.8%]	[47.6%]	[233.3%]

	{178.5%,206.3%}	{35.7%,59.4%}	{219.3%,247.0%}

Age 10-19 years			

Note: Standard errors of effects are in parentheses; the % changes in hospitalizations relative to the unaffected control group due to the cleft effects are listed in brackets (the 95% confidence intervals of these % changes are included in curly brackets); ** = p < 0.05; **** = p < 0.001; the effects are estimated from (Equation 1), adjusting for the covariates in that model.

## Discussion

The study finds significant effects of oral clefts on increasing hospitalizations from birth through age 59 years. The effects are largest during the first 10 years of life and decrease with age after that but may remain large. In the first 10 years of life, oral clefts triple the hospital admission probability (about 195% probability increase), increase length of stay among those hospitalized by about 50%, and triple total hospitalization days relative to controls. Between ages 50 and 59 years, oral clefts increase admission probability by about 13% relative to controls. These effects are mainly driven by cleft lip with palate which has the largest effects in most age groups followed by cleft palate alone, while cleft lip alone has the smallest effects, indicating increasing effects with cleft severity. Cleft lip with palate and cleft palate alone have sizable effects on hospitalizations during both childhood and late adulthood. The largest effects are for cleft lip with palate during the first 19 years of life, where total hospitalizations are increased by more than 330% relative to controls. Cleft lip with palate increases admission probability between 50 and 59 years of age by about 24.5%, while cleft palate alone increases length of stay for those hospitalized by 32% between 40 and 49 years of age. Oral clefts have no adverse effects on hospitalization in the study's oldest age group of 60-68 years. The effects of oral clefts on increasing hospitalization during adulthood (30 years and older) appear to be largely due to oral clefts reducing individual-level socioeconomic performance which in turn increases hospitalization risks.

A particular strength of the study is the large population-level sample of individuals with and without oral clefts, which significantly enhances the generalizability of the results. An additional strength is studying hospitalizations throughout most of the average lifespan - up to 68 years. A third strength is the high-quality data on hospitalizations and other study variables which were collected as part of administrative population-wide registry systems (described above) and are not based on self-report which is subject to recall and report biases. The study analytical sample described in Tables 1 and 2 includes about 4.5 million yearly observations that represent 96% of the total number of observations identified for the study - the remaining 4% had incomplete data on the control variables. As mentioned above, studies of the long-term effects of oral clefts on healthcare use are rare and these data strengths enhance the contribution of the study to the literature. The study results are consistent with previous studies that report significant in-patient healthcare costs during the first 10 years of life for children with clefts using data from the United States [31] as well as higher hospitalization risks due to certain psychiatric conditions among adults with oral clefts in Denmark [32].

The study has some limitations that warrant discussion. The study only includes data on individuals during the years that they were alive and does not account for individuals who have died or migrated out of Denmark and have censored hospitalization outcomes. Differences in mortality and migration between affected and unaffected individuals may have opposite effects on the study results. Oral clefts may increase life-long mortality risks [19]. Individuals who have died and were not included in the analysis would have been expected to have on average larger hospitalization risks and longer length of stay had they remained alive than individuals in the study sample. Furthermore, among individuals who died, those with clefts would have been expected to have more hospitalizations on average than those without clefts had they stayed alive. Therefore, censoring due to mortality might contribute to the underestimation of oral cleft effects on increasing hospital admission and length of stay for the population of affected individuals if oral-cleft mortality risks decrease in the future, particularly for the oldest study age group (60-68 years) for which mortality risks are higher and we find no significant oral cleft effects on hospital use.

In contrast, there is an overall higher rate of migrating out of Denmark for at least two years by about 2-3 percentage points in controls compared to individuals with oral clefts born between 1960 and 1989. Specifically, these migration rates in the control group are 8.1%, 6.7%, 3.4% for birth years 1960-1969, 1970-1979, and 1980-1989, respectively, compared to 5.4%, 4.2% and 1.9% for individuals born with oral clefts in these years, respectively. There are no significant differences in migration rates between cases and controls born in earlier or later years and included in our study. If individuals who migrate out are healthier and have lower hospitalization risks, this may slightly reduce the generalizability of the results and lead to overestimation of the oral cleft effects on hospitalization for the age groups that include the birth cohorts with significant migration differences between affected individuals and controls. However, given the overall small difference in migration rates, it is unlikely that this significantly biases the study results. Furthermore, we do adjust in the model for the number of days when the study subjects were in Denmark in a given year, which accounts for differences in migration between affected individuals and controls for years when individuals were partly present in the country.

Another limitation is that we cannot control in the older study age groups for parental baseline socioeconomic status and demographic factors that may affect cleft risks and child and adult health and hospitalization. However, as mentioned above, we find virtually similar effects of oral clefts on hospitalizations for ages 19 years and younger when we exclude parental socioeconomic characteristics. Therefore, it is unlikely that this limitation has any serious effects on the study results.

Given that the control group is a random sample of all births although it excludes oral cleft cases, some controls also have non-cleft birth defects. This is expected to result in underestimation of the oral cleft effects on hospitalization. It is impossible to identify individuals with birth defects from the control sample who were born before 1977. However, we are able to identify from the National Patient Registry individuals in the control sample who were born in 1977 and after and who have been diagnosed with a birth defect within the first three years of life. As expected and shown in Table 6, excluding these individuals decreases the hospitalization rates and average length of stay among the control group and provides further support for considering the oral cleft effects estimated in this study to be lower bounds for the real effects.

**Table 6:** Hospitalization Rates in the Control Sample

	0-9 years of age		10-19 years of age	
	**Including controls with malformations**	**Excluding controls with malformations**	**Including controls with malformations**	**Excluding controls with malformations**

Number of observations	618,082	586,874	343,797	326,991

Hospitalized at least once	54,562 (8.83%)	48,280 (8.23%)	16,967 (4.94%)	15,791 (4.83%)

Hospitalization days among those hospitalized	5.23 (11.37)	4.68 (9.73)	3.88 (9.27)	3.83 (9.39)

Note: The Table reports crude (unadjusted) hospitalization rates and average length of stay in the control sample born between 1977 and 2002 when including and excluding individuals with birth defects. The frequency of those hospitalized is reported with the percentage in parenthesis. The mean of hospitalization days is reported with the standard deviation in parenthesis.

Finally, it is possible that multiple testing has increased Type 1 error. This combined with the large sample we analyze may have increased the statistical significance of the results. However, we limit the number of statistical tests for oral cleft effects in order to reduce the effect of multiple testing. Furthermore, several oral cleft effects are significant at p < 0.001, the significance threshold from a Bonferroni correction for 50 tests, which exceeds the number of tests that we conducted. We view the use of a large sample as a strength not just for enhancing the generalizability of the study results but also for increasing the study power to detect small to moderate effects that may not be detected in smaller samples but are still of clinical relevance. The effects of oral clefts on hospitalizations that we find in this study are small to moderate in magnitude, which adds validity to them as they are more believable than large effects. However, these effects are still clinically important especially during childhood and adolescence. For example, a child born with cleft lip with palate will have on average 25 more hospitalization days by age 19 years compared to an unaffected child. The validity and significance of the results as a whole are unlikely to have been affected by Type 1 error inflation.

## Conclusions

In conclusion, we find that individuals with oral clefts use hospital care more than unaffected individuals beginning in early childhood through adulthood. This increase in hospitalizations is largest during the first 10 years of life and is more pronounced for individuals with cleft lip with palate. The study has important implications for improving the care of individuals with oral clefts and for healthcare policymaking. The increased hospital use with oral clefts over most of the average lifespan emphasizes the importance of acknowledging oral clefts as lifelong morbidity risk factors with health burdens beyond infancy and childhood. Optimizing the wellbeing of affected individuals requires treatment programs that account for the above-average hospitalization risks throughout life and provide preventive interventions to reduce these risks. The increased hospitalization risks suggest that individuals with oral clefts may have greater need for healthcare insurance than unaffected individuals. This highlights the importance of policies that enhance the access of affected individuals to insurance in countries where individuals with birth defects face larger barriers to insurance than the general population because of pre-existing condition exclusions. Furthermore, improving the long-term health of individuals with oral clefts and reducing their hospitalization risks involves reducing the adverse effects of oral clefts on psychosocial and economic performance outcomes such as marriage, education, employment, and income. This highlights the importance of evaluating the costs and benefits of interventions aimed at enhancing investments in the human capital of affected individuals.

The study highlights several relevant questions for future studies. One question is identifying the health problems among affected individuals that result in increased hospitalizations and common etiologies as this will be important for identifying prevention strategies and improving health outcomes. Another question is assessing the interactions between oral cleft status and family socioeconomic and demographic backgrounds that may modify the oral cleft effects on hospitalizations in order to identify groups at higher risks for hospitalizations who may benefit more from focused interventions. There are virtually no differences in the supply of and access to providers of oral cleft repair surgeries in Denmark given that these surgeries have been centralized at two hospitals since the mid 1930s. Also, there is limited variation in cleft repair surgery take-up and timing due to the universal healthcare system. Therefore, variations in supply and quality of cleft repair surgery and take-up of these surgeries do not explain the study findings. Nonetheless, in countries where such variations exist such as the United States, evaluating how they affect long-term hospital use of affected individuals is needed. Finally, the study highlights the importance of studying the long-term effects of oral clefts on other types of healthcare use including outpatient, emergency, and dental care.

## Competing interests

The authors declare that they do not have any financial or other competing interests in this work.

## Authors' contributions

Drs. Wehby, Christensen, and Murray conceived the study. Dr. Wehby designed the study models and statistical analysis in collaboration with all co-authors. Mrs. Almind Pedersen implemented the statistical analysis. Dr. Wehby wrote the first draft. All co-authors contributed significantly to writing and critically revising the manuscript.

## Pre-publication history

The pre-publication history for this paper can be accessed here:

http://www.biomedcentral.com/1472-6963/12/58/prepub

## Supplementary Material

Additional file 1 **Table S1**. Detailed Logistic and Poisson Regression Results for Age Group 0-9 years. Click here for file

Additional file 2 **Table S2**. Detailed Logistic and Poisson Regression Results for Age Group 10-19 years. Click here for file

Additional file 3 **Table S3**. Detailed Logistic and Poisson Regression Results for Age Group 20-29 years. Click here for file

Additional file 4 **Table S4**. Detailed Logistic and Poisson Regression Results for Age Group 30-39 years. Click here for file

Additional file 5 **Table S5**. Detailed Logistic and Poisson Regression Results for Age Group 40-49 years. Click here for file

Additional file 6 **Table S6**. Detailed Logistic and Poisson Regression Results for Age Group 50-59 years. Click here for file

Additional file 7 **Table S7**. Detailed Logistic and Poisson Regression Results for Age Group 60-68 years. Click here for file
