# Supplementary material for: The effects of oral clefts on hospital use throughout the lifespan
Source: BMC Health Serv Res. 2012 Mar 9;12:58. doi: 10.1186/1472-6963-12-58 (PMC3350419; doi:10.1186/1472-6963-12-58)
Supplement: Additional file 1 — Table S1. Detailed Logistic and Poisson Regression Results for Age Group 0-9 years. [file 1472-6963-12-58-S1.DOC]

Table S1: Detailed Logistic and Poisson Regression Results for Age Group 0-9 years

|  | Logistic regression | | | | Poisson regression | | | |
| --- | --- | --- | --- | --- | --- | --- | --- | --- |
|  | Full Model | | Excluding Parental SES Characteristics | | Full Model | | Excluding Parental SES Characteristics | |
|  | *Any cleft model* | *Cleft types model* | *Any cleft model* | *Cleft types model* | *Any cleft model* | *Cleft types model* | *Any cleft model* | *Cleft types model* |
| Cleft Status | 1.38^****^ |  | 1.382^****^ |  | 0.41^****^ |  | 0.419^****^ |  |
|  | (0.02) |  | (0.017) |  | (0.02) |  | (0.024) |  |
| Cleft lip |  | 0.94^****^ |  | 0.925^****^ |  | 0.21^****^ |  | 0.212^****^ |
|  |  | (0.03) |  | (0.028) |  | (0.03) |  | (0.032) |
| Cleft lip with palate |  | 1.74^****^ |  | 1.742^****^ |  | 0.52^****^ |  | 0.521^****^ |
|  |  | (0.02) |  | (0.023) |  | (0.03) |  | (0.034) |
| Cleft palate |  | 1.30^****^ |  | 1.332^****^ |  | 0.39^****^ |  | 0.399^****^ |
|  |  | (0.03) |  | (0.033) |  | (0.04) |  | (0.043) |
| Male | 0.33^****^ | 0.32^****^ | 0.325^****^ | 0.322^****^ | -0.04^*^ | -0.04^*^ | -0.039^*^ | -0.040^*^ |
|  | (0.01) | (0.01) | (0.011) | (0.011) | (0.02) | (0.02) | (0.021) | (0.021) |
| Age (years) | -0.19^****^ | -0.19^****^ | -0.192^****^ | -0.192^****^ | -0.09^****^ | -0.09^****^ | -0.077^****^ | -0.077^****^ |
|  | (0.002) | (0.002) | (0.002) | (0.002) | (0.01) | (0.01) | (0.005) | (0.005) |
| Exposure time (days) | 0.00007 | 0.00007 | 0.00002 | 0.00003 | -0.002^****^ | -0.002^****^ | -0.002^****^ | -0.002^****^ |
|  | (0.00006) | (0.00006) | (0.00006) | (0.00006) | (0.00009) | (0.00009) | (0.00009) | (0.00009) |
| Maternal age (years) | -0.002 | -0.002 |  |  | 0.01^***^ | 0.01^***^ |  |  |
|  | (0.002) | (0.002) |  |  | (0.003) | (0.003) |  |  |
| Paternal age (years) | -0.0007 | -0.0007 |  |  | 0.003 | 0.003 |  |  |
|  | (0.001) | (0.001) |  |  | (0.002) | (0.002) |  |  |
| Maternal upper and post-secondary | -0.11^****^ | -0.11^****^ |  |  | -0.05^**^ | -0.05^**^ |  |  |
|  | (0.01) | (0.01) |  |  | (0.03) | (0.03) |  |  |
| Maternal tertiary | -0.16^****^ | -0.15^****^ |  |  | -0.06^*^ | -0.06^*^ |  |  |
|  | (0.02) | (0.02) |  |  | (0.03) | (0.03) |  |  |
| Paternal upper and post-secondary | -0.05^****^ | -0.05^****^ |  |  | -0.04^*^ | -0.05^*^ |  |  |
|  | (0.01) | (0.01) |  |  | (0.03) | (0.03) |  |  |
| Paternal tertiary | -0.12^****^ | -0.12^****^ |  |  | -0.08^**^ | -0.08^**^ |  |  |
|  | (0.02) | (0.02) |  |  | (0.03) | (0.03) |  |  |
| Maternal income quintile  20-40% | -0.05^***^ | -0.04^***^ |  |  | -0.12^****^ | -0.12^****^ |  |  |
|  | (0.02) | (0.02) |  |  | (0.03) | (0.03) |  |  |
| Maternal income quintile  40-60% | -0.05^***^ | -0.04^***^ |  |  | -0.11^***^ | -0.11^***^ |  |  |
|  | (0.02) | (0.02) |  |  | (0.03) | (0.03) |  |  |
| Maternal income quintile  60-80% | -0.07^****^ | -0.06^****^ |  |  | -0.15^****^ | -0.15*^***^ |  |  |
|  | (0.02) | (0.02) |  |  | (0.03) | (0.03) |  |  |
| Maternal income quintile  80-100% | -0.04^**^ | -0.04^**^ |  |  | -0.18^****^ | -0.18^****^ |  |  |
|  | (0.02) | (0.02) |  |  | (0.04) | (0.04) |  |  |
| Paternal income quintile  20-40% | -0.02 | -0.02 |  |  | 0.03 | 0.04 |  |  |
|  | (0.02) | (0.02) |  |  | (0.03) | (0.03) |  |  |
| Paternal income quintile  40-60% | -0.04^**^ | -0.04^**^ |  |  | 0.01 | 0.005 |  |  |
|  | (0.02) | (0.02) |  |  | (0.03) | (0.03) |  |  |
| Paternal income quintile  60-80% | -0.07^****^ | -0.07^****^ |  |  | -0.04 | -0.04 |  |  |
|  | (0.02) | (0.02) |  |  | (0.03) | (0.03) |  |  |
| Paternal income quintile  80-100% | -0.09^****^ | -0.08^****^ |  |  | -0.09^***^ | -0.09^**^ |  |  |
|  | (0.02) | (0.02) |  |  | (0.03) | (0.03) |  |  |
| Maternal Employed | 0.06^**^ | 0.06^**^ |  |  | -0.07 | -0.07 |  |  |
|  | (0.03) | (0.03) |  |  | (0.07) | (0.07) |  |  |
| Maternal Unemployed/other | 0.13^****^ | 0.14^****^ |  |  | -0.09 | -0.08 |  |  |
|  | (0.03) | (0.03) |  |  | (0.07) | (0.07) |  |  |
| Paternal Employed | 0.04^**^ | 0.04^**^ |  |  | -0.01 | -0.01 |  |  |
|  | (0.02) | (0.02) |  |  | (0.04) | (0.04) |  |  |
| Paternal Unemployed/other | 0.08^****^ | 0.08^****^ |  |  | 0.07 | 0.07 |  |  |
|  | (0.02) | (0.02) |  |  | (0.05) | (0.05) |  |  |
| Cohabiting | 0.02 | 0.02 |  |  | 0.01 | 0.01 |  |  |
|  | (0.01) | (0.01) |  |  | (0.02) | (0.02) |  |  |
| Single | 0.14^****^ | 0.14^****^ |  |  | 0.05 | 0.05 |  |  |
|  | (0.02) | (0.02) |  |  | (0.03) | (0.03) |  |  |
| 500-999 Inh/km2 | -0.01 | -0.01 |  |  | -0.04 | -0.04 |  |  |
|  | (0.03) | (0.03) |  |  | (0.05) | (0.05) |  |  |
| 200-499 Inh/km2 | -0.04 | -0.05 |  |  | -0.12^*^ | -0.13^*^ |  |  |
|  | (0.04) | (0.04) |  |  | (0.07) | (0.07) |  |  |
| 100-199 Inh/km2 | -0.03 | -0.03 |  |  | -0.06 | -0.07 |  |  |
|  | (0.04) | (0.04) |  |  | (0.07) | (0.07) |  |  |
| 50-99 Inh/km2 | -0.09^**^ | -0.09^**^ |  |  | -0.05 | -0.05 |  |  |
|  | (0.04) | (0.04) |  |  | (0.07) | (0.07) |  |  |
| <50 Inh/km2 | -0.11^***^ | -0.11^***^ |  |  | -0.10 | -0.10 |  |  |
|  | (0.04) | (0.04) |  |  | (0.07) | (0.07) |  |  |
| Constant | -1.42^****^ | -1.41^****^ | -1.728^****^ | -1.724^****^ | 2.39^****^ | 2.40^****^ | 2.475^****^ | 2.483^****^ |
|  | (0.06) | (0.06) | (0.027) | (0.027) | (0.12) | (0.12) | (0.043) | (0.043) |
| Observations | 691766 | 691766 | 691766 | 691766 | 64236 | 64236 | 64236 | 64236 |

Note: The Table reports the regression coefficients and their standard errors in parentheses; *=p<1; **=p<0.05; ***=p<0.01; ****=p<0.001; results for county and year binary indicators are omitted for brevity.
