# Supplementary material for: The effects of oral clefts on hospital use throughout the lifespan
Source: BMC Health Serv Res. 2012 Mar 9;12:58. doi: 10.1186/1472-6963-12-58 (PMC3350419; doi:10.1186/1472-6963-12-58)
Supplement: Additional file 2 — Table S2. Detailed Logistic and Poisson Regression Results for Age Group 10-19 years. [file 1472-6963-12-58-S2.DOC]

Table S2: Detailed Logistic and Poisson Regression Results for Age Group 10-19 years

|  | Logistic regression | | | | Poisson regression | | | |
| --- | --- | --- | --- | --- | --- | --- | --- | --- |
|  | Full Model | | Excluding Parental SES Characteristics | | Full Model | | Excluding Parental SES Characteristics | |
|  | *Any cleft model* | *Cleft types model* | *Any cleft model* | *Cleft types model* | *Any cleft model* | *Cleft types model* | *Any cleft model* | *Cleft types model* |
| Cleft Status | 1.18^****^ |  | 1.202^****^ |  | 0.21^****^ |  | 0.208^****^ |  |
|  | (0.02) |  | (0.022) |  | (0.02) |  | (0.024) |  |
| Cleft lip |  | 0.61^****^ |  | 0.627^****^ |  | 0.06 |  | 0.055 |
|  |  | (0.04) |  | (0.043) |  | (0.04) |  | (0.043) |
| Cleft lip with palate |  | 1.79^****^ |  | 1.802^****^ |  | 0.28^****^ |  | 0.276^****^ |
|  |  | (0.03) |  | (0.025) |  | (0.03) |  | (0.026) |
| Cleft palate |  | 0.74^****^ |  | 0.771^****^ |  | 0.13^**^ |  | 0.137^**^ |
|  |  | (0.05) |  | (0.046) |  | (0.05) |  | (0.055) |
| Male | -0.09^****^ | -0.10^****^ | -0.093^****^ | -0.106^****^ | -0.03 | -0.03 | -0.023 | -0.026 |
|  | (0.01) | (0.01) | (0.012) | (0.012) | (0.03) | (0.03) | (0.026) | (0.026) |
| Age (years) | 0.07^****^ | 0.08^****^ | 0.064^****^ | 0.064^****^ | 0.01^***^ | 0.01^***^ | 0.014^****^ | 0.014^****^ |
|  | (0.002) | (0.002) | (0.002) | (0.002) | (0.005) | (0.005) | (0.004) | (0.004) |
| Exposure time (days) | -0.002^****^ | -0.002^****^ | -0.002^****^ | -0.002^****^ | -0.003^****^ | -0.003^****^ | -0.003^****^ | -0.003^****^ |
|  | (0.0004) | (0.0004) | (0.0004) | (0.0004) | (0.0006) | (0.0006) | (0.001) | (0.001) |
| Maternal age (years) | -0.01^****^ | -0.01^****^ |  |  | -0.001 | -0.001 |  |  |
|  | (0.002) | (0.002) |  |  | (0.004) | (0.004) |  |  |
| Paternal age (years) | -0.003^**^ | -0.003^**^ |  |  | 0.0008 | 0.0008 |  |  |
|  | (0.002) | (0.002) |  |  | (0.003) | (0.003) |  |  |
| Maternal upper and post-secondary | -0.10^****^ | -0.09^****^ |  |  | -0.004 | -0.004 |  |  |
|  | (0.01) | (0.01) |  |  | (0.03) | (0.03) |  |  |
| Maternal tertiary | -0.16^****^ | -0.16^****^ |  |  | 0.07 | 0.07 |  |  |
|  | (0.02) | (0.02) |  |  | (0.04) | (0.04) |  |  |
| Paternal upper and post-secondary | -0.04^***^ | -0.04^***^ |  |  | -0.02 | -0.02 |  |  |
|  | (0.01) | (0.01) |  |  | (0.03) | (0.03) |  |  |
| Paternal tertiary | -0.09^****^ | -0.09^****^ |  |  | -0.02 | -0.02 |  |  |
|  | (0.02) | (0.02) |  |  | (0.04) | (0.04) |  |  |
| Maternal income quintile  20-40% | 0.01 | 0.01 |  |  | -0.06^*^ | -0.06^*^ |  |  |
|  | (0.02) | (0.02) |  |  | (0.04) | (0.04) |  |  |
| Maternal income quintile  40-60% | 0.01 | 0.01 |  |  | -0.07^*^ | -0.07^*^ |  |  |
|  | (0.02) | (0.02) |  |  | (0.04) | (0.04) |  |  |
| Maternal income quintile  60-80% | 0.03 | 0.03 |  |  | -0.10^**^ | -0.10^**^ |  |  |
|  | (0.02) | (0.02) |  |  | (0.04) | (0.04) |  |  |
| Maternal income quintile  80-100% | 0.03 | 0.04 |  |  | -0.15^****^ | -0.15^****^ |  |  |
|  | (0.02) | (0.02) |  |  | (0.04) | (0.04) |  |  |
| Paternal income quintile  20-40% | -0.08^****^ | -0.08^****^ |  |  | 0.002 | 0.002 |  |  |
|  | (0.02) | (0.02) |  |  | (0.03) | (0.03) |  |  |
| Paternal income quintile  40-60% | -0.10^****^ | -0.10^****^ |  |  | -0.02 | -0.02 |  |  |
|  | (0.02) | (0.02) |  |  | (0.04) | (0.04) |  |  |
| Paternal income quintile  60-80% | -0.10^****^ | -0.10^****^ |  |  | -0.05 | -0.05 |  |  |
|  | (0.02) | (0.02) |  |  | (0.04) | (0.04) |  |  |
| Paternal income quintile  80-100% | -0.12^****^ | -0.12^****^ |  |  | -0.05 | -0.05 |  |  |
|  | (0.02) | (0.02) |  |  | (0.04) | (0.04) |  |  |
| Maternal Employed | 0.02 | 0.02 |  |  | 0.08^**^ | 0.08^**^ |  |  |
|  | (0.03) | (0.03) |  |  | (0.04) | (0.04) |  |  |
| Maternal Unemployed/other | 0.18^****^ | 0.18^****^ |  |  | 0.11^**^ | 0.11^**^ |  |  |
|  | (0.03) | (0.03) |  |  | (0.04) | (0.04) |  |  |
| Paternal Employed | 0.05^***^ | 0.05^***^ |  |  | -0.03 | -0.03 |  |  |
|  | (0.02) | (0.02) |  |  | (0.03) | (0.03) |  |  |
| Paternal Unemployed/other | 0.13^****^ | 0.14^****^ |  |  | -0.02 | -0.02 |  |  |
|  | (0.03) | (0.03) |  |  | (0.05) | (0.05) |  |  |
| Cohabiting | 0.11^****^ | 0.12^****^ |  |  | -0.01 | -0.01 |  |  |
|  | (0.02) | (0.02) |  |  | (0.05) | (0.05) |  |  |
| Single | 0.15^****^ | 0.15^****^ |  |  | -0.03 | -0.03 |  |  |
|  | (0.02) | (0.02) |  |  | (0.03) | (0.03) |  |  |
| 500-999 Inh/km2 | 0.01 | 0.02 |  |  | 0.01 | 0.01 |  |  |
|  | (0.04) | (0.04) |  |  | (0.09) | (0.09) |  |  |
| 200-499 Inh/km2 | 0.05 | 0.05 |  |  | -0.05 | -0.05 |  |  |
|  | (0.04) | (0.04) |  |  | (0.10) | (0.10) |  |  |
| 100-199 Inh/km2 | 0.04 | 0.04 |  |  | 0.04 | 0.05 |  |  |
|  | (0.05) | (0.05) |  |  | (0.10) | (0.10) |  |  |
| 50-99 Inh/km2 | 0.02 | 0.02 |  |  | -0.002 | 0.00007 |  |  |
|  | (0.04) | (0.04) |  |  | (0.10) | (0.10) |  |  |
| <50 Inh/km2 | 0.02 | 0.02 |  |  | -0.07 | -0.07 |  |  |
|  | (0.05) | (0.05) |  |  | (0.10) | (0.10) |  |  |
| Constant | -2.56^****^ | -2.53^****^ | -3.068^****^ | -3.063^****^ | 2.71^****^ | 2.72^****^ | 2.624^****^ | 2.634^****^ |
|  | (0.16) | (0.16) | (0.156) | (0.157) | (0.25) | (0.25) | (0.217) | (0.217) |
| Observations | 710815 | 710815 | 710815 | 710815 | 42787 | 42787 | 42787 | 42787 |

Note: The Table reports the regression coefficients and their standard errors in parentheses; *=p<1; **=p<0.05; ***=p<0.01; ****=p<0.001; results for county and year binary indicators are omitted for brevity
