# Supplementary material for: The effects of oral clefts on hospital use throughout the lifespan
Source: BMC Health Serv Res. 2012 Mar 9;12:58. doi: 10.1186/1472-6963-12-58 (PMC3350419; doi:10.1186/1472-6963-12-58)
Supplement: Additional file 3 — Table S3. Detailed Logistic and Poisson Regression Results for Age Group 20-29 years. [file 1472-6963-12-58-S3.DOC]

Table S3: Detailed Logistic and Poisson Regression Results for Age Group 20-29 years

|  | Logistic regression | | | Poisson regression | | |
| --- | --- | --- | --- | --- | --- | --- |
|  | Full Model | | Excluding Own SES Characteristics | Full Model | | Excluding Own SES Characteristics |
|  | *Any cleft model* | *Cleft types model* | *Any cleft model* | *Any cleft model* | *Cleft types model* | *Any cleft model* |
| Cleft Status | 0.360^****^ |  | 0.365^****^ | 0.079^**^ |  | 0.094^***^ |
|  | (0.023) |  | (0.023) | (0.034) |  | (0.034) |
| Cleft lip |  | 0.142^***^ |  |  | 0.057 |  |
|  |  | (0.042) |  |  | (0.095) |  |
| Cleft lip with palate |  | 0.714^****^ |  |  | 0.066^**^ |  |
|  |  | (0.035) |  |  | (0.030) |  |
| Cleft palate |  | 0.132^***^ |  |  | 0.113^*^ |  |
|  |  | (0.041) |  |  | (0.062) |  |
| Male | -1.163^****^ | -1.167^****^ | -1.262^****^ | -0.008 | -0.007 | 0.007 |
|  | (0.010) | (0.010) | (0.010) | (0.022) | (0.022) | (0.022) |
| Age (years) | 0.032^****^ | 0.032^****^ | 0.071^****^ | 0.015^****^ | 0.015^****^ | 0.006^**^ |
|  | (0.002) | (0.002) | (0.001) | (0.003) | (0.003) | (0.002) |
| Exposure time (days) | 0.002^****^ | 0.002^****^ | 0.003^****^ | -0.002^****^ | -0.002^****^ | -0.002^****^ |
|  | (0.0002) | (0.0003) | (0.0003) | (0.0004) | (0.0004) | (0.0004) |
| Upper and post-secondary | -0.375^****^ | -0.375^****^ |  | -0.060^****^ | -0.060^****^ |  |
|  | (0.009) | (0.009) |  | (0.017) | (0.017) |  |
| Tertiary | -0.295^****^ | -0.295^****^ |  | -0.120^****^ | -0.119^****^ |  |
|  | (0.014) | (0.014) |  | (0.022) | (0.022) |  |
| Income quintile  20-40% | 0.091^****^ | 0.091^****^ |  | 0.052^**^ | 0.052^**^ |  |
|  | (0.013) | (0.013) |  | (0.026) | (0.026) |  |
| Income quintile  40-60% | 0.270^****^ | 0.270^****^ |  | 0.009 | 0.009 |  |
|  | (0.013) | (0.013) |  | (0.024) | (0.024) |  |
| Income quintile  60-80% | 0.358^****^ | 0.358^****^ |  | 0.050^*^ | 0.050^*^ |  |
|  | (0.014) | (0.014) |  | (0.026) | (0.026) |  |
| Income quintile  80-100% | 0.197^****^ | 0.197^****^ |  | 0.030 | 0.030 |  |
|  | (0.017) | (0.017) |  | (0.030) | (0.030) |  |
| Employed | 0.065^**^ | 0.064^*^ |  | -0.008 | -0.008 |  |
|  | (0.033) | (0.033) |  | (0.048) | (0.048) |  |
| Unemployed/other | 0.346^****^ | 0.346^****^ |  | 0.134^***^ | 0.133^***^ |  |
|  | (0.033) | (0.033) |  | (0.049) | (0.049) |  |
| Cohabiting | -0.357^****^ | -0.357^****^ |  | 0.026^*^ | 0.026^*^ |  |
|  | (0.011) | (0.011) |  | (0.016) | (0.016) |  |
| Single | -0.718^****^ | -0.719^****^ |  | 0.069^****^ | 0.069^****^ |  |
|  | (0.012) | (0.012) |  | (0.020) | (0.020) |  |
| 500-999 Inh/km2 | -0.001 | -0.001 |  | -0.029 | -0.029 |  |
|  | (0.028) | (0.028) |  | (0.047) | (0.047) |  |
| 200-499 Inh/km2 | 0.108^***^ | 0.109^***^ |  | -0.095^*^ | -0.094^*^ |  |
|  | (0.033) | (0.033) |  | (0.056) | (0.057) |  |
| 100-199 Inh/km2 | 0.167^****^ | 0.167^****^ |  | -0.062 | -0.061 |  |
|  | (0.033) | (0.033) |  | (0.057) | (0.057) |  |
| 50-99 Inh/km2 | 0.180^****^ | 0.180^****^ |  | -0.092^*^ | -0.091^*^ |  |
|  | (0.033) | (0.033) |  | (0.055) | (0.055) |  |
| <50 Inh/km2 | 0.230^****^ | 0.231^****^ |  | -0.069 | -0.069 |  |
|  | (0.034) | (0.034) |  | (0.057) | (0.057) |  |
| Constant | -2.890^****^ | -2.887^****^ | -4.238^****^ | 2.200^****^ | 2.200^****^ | 2.538^****^ |
|  | (0.108) | (0.108) | (0.099) | (0.160) | (0.160) | (0.149) |
| Observations | 840639 | 840639 | 840639 | 98793 | 98793 | 98793 |

Note: The Table reports the regression coefficients and their standard errors in parentheses; *=p<1; **=p<0.05; ***=p<0.01; ****=p<0.001; results for county and year binary indicators are omitted for brevity
