# Supplementary material for: The effects of oral clefts on hospital use throughout the lifespan
Source: BMC Health Serv Res. 2012 Mar 9;12:58. doi: 10.1186/1472-6963-12-58 (PMC3350419; doi:10.1186/1472-6963-12-58)
Supplement: Additional file 4 — Table S4. Detailed Logistic and Poisson Regression Results for Age Group 30-39 years. [file 1472-6963-12-58-S4.DOC]

Table S4: Detailed Logistic and Poisson Regression Results for Age Group 30-39 years

|  | Logistic regression | | | Poisson regression | | |
| --- | --- | --- | --- | --- | --- | --- |
|  | Full Model | | Excluding Own SES Characteristics | Full Model | | Excluding Own SES Characteristics |
|  | *Any cleft model* | *Cleft types model* | *Any cleft model* | *Any cleft model* | *Cleft types model* | *Any cleft model* |
| Cleft Status | 0.161^****^ |  | 0.178^****^ | 0.038 |  | 0.086^*^ |
|  | (0.025) |  | (0.025) | (0.043) |  | (0.044) |
| Cleft lip |  | 0.074^*^ |  |  | 0.020 |  |
|  |  | (0.044) |  |  | (0.067) |  |
| Cleft lip with palate |  | 0.357^****^ |  |  | 0.038 |  |
|  |  | (0.039) |  |  | (0.068) |  |
| Cleft palate |  | 0.006 |  |  | 0.055 |  |
|  |  | (0.045) |  |  | (0.086) |  |
| Male | -1.004^****^ | -1.007^****^ | -1.071^****^ | 0.115^****^ | 0.115^****^ | 0.127^****^ |
|  | (0.011) | (0.011) | (0.010) | (0.021) | (0.021) | (0.019) |
| Age (years) | -0.057^****^ | -0.057^****^ | -0.060^****^ | 0.016^****^ | 0.016^****^ | 0.016^****^ |
|  | (0.001) | (0.001) | (0.001) | (0.002) | (0.002) | (0.002) |
| Exposure time (days) | -0.002^****^ | -0.002^****^ | -0.002^****^ | -0.003^****^ | -0.003^****^ | -0.004^****^ |
|  | (0.0002) | (0.0002) | (0.0002) | (0.0002) | (0.0002) | (0.0002) |
| Upper and post-secondary | -0.093^****^ | -0.093^****^ |  | -0.053^***^ | -0.053^***^ |  |
|  | (0.011) | (0.011) |  | (0.019) | (0.019) |  |
| Tertiary | 0.003 | 0.003 |  | -0.075^***^ | -0.075^***^ |  |
|  | (0.012) | (0.012) |  | (0.022) | (0.022) |  |
| Income quintile  20-40% | 0.021^*^ | 0.021^*^ |  | -0.034^*^ | -0.034^*^ |  |
|  | (0.012) | (0.012) |  | (0.019) | (0.019) |  |
| Income quintile  40-60% | 0.050^****^ | 0.050^****^ |  | -0.016 | -0.016 |  |
|  | (0.013) | (0.013) |  | (0.024) | (0.024) |  |
| Income quintile  60-80% | -0.016 | -0.016 |  | -0.079^***^ | -0.079^***^ |  |
|  | (0.015) | (0.015) |  | (0.026) | (0.026) |  |
| Income quintile  80-100% | -0.123^****^ | -0.122^****^ |  | -0.142^****^ | -0.141^****^ |  |
|  | (0.017) | (0.017) |  | (0.029) | (0.029) |  |
| Employed | 0.059^***^ | 0.059^***^ |  | -0.039 | -0.040 |  |
|  | (0.020) | (0.020) |  | (0.035) | (0.035) |  |
| Unemployed/other | 0.370^****^ | 0.370^****^ |  | 0.148^****^ | 0.148^****^ |  |
|  | (0.022) | (0.022) |  | (0.037) | (0.037) |  |
| Cohabiting | 0.126^****^ | 0.126^****^ |  | 0.077^****^ | 0.077^****^ |  |
|  | (0.010) | (0.010) |  | (0.018) | (0.018) |  |
| Single | -0.076^****^ | -0.076^****^ |  | 0.203^****^ | 0.203^****^ |  |
|  | (0.011) | (0.011) |  | (0.020) | (0.020) |  |
| 500-999 Inh/km2 | 0.011 | 0.012 |  | -0.015 | -0.015 |  |
|  | (0.028) | (0.028) |  | (0.042) | (0.042) |  |
| 200-499 Inh/km2 | 0.029 | 0.030 |  | -0.046 | -0.046 |  |
|  | (0.032) | (0.032) |  | (0.052) | (0.052) |  |
| 100-199 Inh/km2 | 0.047 | 0.047 |  | -0.045 | -0.045 |  |
|  | (0.033) | (0.033) |  | (0.054) | (0.054) |  |
| 50-99 Inh/km2 | 0.024 | 0.025 |  | -0.062 | -0.062 |  |
|  | (0.032) | (0.032) |  | (0.051) | (0.051) |  |
| <50 Inh/km2 | 0.042 | 0.043 |  | -0.062 | -0.062 |  |
|  | (0.034) | (0.034) |  | (0.055) | (0.055) |  |
| Constant | 0.864^****^ | 0.866^****^ | 1.162^****^ | 2.624^****^ | 2.624^****^ | 2.743^****^ |
|  | (0.088) | (0.088) | (0.083) | (0.131) | (0.131) | (0.122) |
| Observations | 872006 | 872006 | 872006 | 96840 | 96840 | 96840 |

Note: The Table reports the regression coefficients and their standard errors in parentheses; *=p<1; **=p<0.05; ***=p<0.01; ****=p<0.001; results for county and year binary indicators are omitted for brevity.
