# Supplementary material for: The effects of oral clefts on hospital use throughout the lifespan
Source: BMC Health Serv Res. 2012 Mar 9;12:58. doi: 10.1186/1472-6963-12-58 (PMC3350419; doi:10.1186/1472-6963-12-58)
Supplement: Additional file 5 — Table S5. Detailed Logistic and Poisson Regression Results for Age Group 40-49 years. [file 1472-6963-12-58-S5.DOC]

Table S5: Detailed Logistic and Poisson Regression Results for Age Group 40-49 years

|  | Logistic regression | | | Poisson regression | | |
| --- | --- | --- | --- | --- | --- | --- |
|  | Full Model | | Excluding Own SES Characteristics | Full Model | | Excluding Own SES Characteristics |
|  | *Any cleft model* | *Cleft types model* | *Any cleft model* | *Any cleft model* | *Cleft types model* | *Any cleft model* |
| Cleft Status | 0.109^***^ |  | 0.197^****^ | 0.045 |  | 0.094^*^ |
|  | (0.032) |  | (0.032) | (0.049) |  | (0.051) |
| Cleft lip |  | -0.060 |  |  | 0.053 |  |
|  |  | (0.058) |  |  | (0.084) |  |
| Cleft lip with palate |  | 0.230^****^ |  |  | -0.088 |  |
|  |  | (0.050) |  |  | (0.064) |  |
| Cleft palate |  | 0.107^*^ |  |  | 0.198^**^ |  |
|  |  | (0.056) |  |  | (0.099) |  |
| Male | -0.189^****^ | -0.190^****^ | -0.317^****^ | 0.109^****^ | 0.111^****^ | 0.070^****^ |
|  | (0.013) | (0.013) | (0.011) | (0.020) | (0.020) | (0.018) |
| Age (years) | 0.007^****^ | 0.006^****^ | 0.008^****^ | 0.026^****^ | 0.026^****^ | 0.028^****^ |
|  | (0.002) | (0.002) | (0.002) | (0.003) | (0.003) | (0.003) |
| Exposure time (days) | -0.007^****^ | -0.007^****^ | -0.008^****^ | -0.003^****^ | -0.003^****^ | -0.004^****^ |
|  | (0.0002) | (0.0002) | (0.0002) | (0.0002) | (0.0001) | (0.0001) |
| Upper and post-secondary | -0.099^****^ | -0.099^****^ |  | -0.002 | -0.002 |  |
|  | (0.013) | (0.013) |  | (0.020) | (0.020) |  |
| Tertiary | -0.189^****^ | -0.188^****^ |  | -0.036 | -0.035 |  |
|  | (0.016) | (0.016) |  | (0.028) | (0.028) |  |
| Income quintile  20-40% | -0.055^****^ | -0.055^****^ |  | -0.035 | -0.035 |  |
|  | (0.015) | (0.015) |  | (0.023) | (0.023) |  |
| Income quintile  40-60% | -0.118^****^ | -0.118^****^ |  | -0.074^***^ | -0.074^***^ |  |
|  | (0.017) | (0.017) |  | (0.026) | (0.026) |  |
| Income quintile  60-80% | -0.228^****^ | -0.228^****^ |  | -0.129^****^ | -0.129^****^ |  |
|  | (0.019) | (0.019) |  | (0.029) | (0.029) |  |
| Income quintile  80-100% | -0.340^****^ | -0.340^****^ |  | -0.186^****^ | -0.186^****^ |  |
|  | (0.021) | (0.021) |  | (0.033) | (0.033) |  |
| Employed | 0.070^****^ | 0.070^****^ |  | 0.046 | 0.046 |  |
|  | (0.019) | (0.019) |  | (0.029) | (0.029) |  |
| Unemployed/other | 0.558^****^ | 0.558^****^ |  | 0.347^****^ | 0.347^****^ |  |
|  | (0.022) | (0.022) |  | (0.034) | (0.034) |  |
| Cohabiting | 0.135^****^ | 0.135^****^ |  | -0.009 | -0.009 |  |
|  | (0.017) | (0.017) |  | (0.028) | (0.028) |  |
| Single | 0.207^****^ | 0.207^****^ |  | 0.148^****^ | 0.148^****^ |  |
|  | (0.013) | (0.013) |  | (0.020) | (0.020) |  |
| 500-999 Inh/km2 | -0.002 | -0.002 |  | 0.013 | 0.013 |  |
|  | (0.035) | (0.035) |  | (0.052) | (0.051) |  |
| 200-499 Inh/km2 | -0.001 | -0.001 |  | 0.018 | 0.019 |  |
|  | (0.040) | (0.040) |  | (0.063) | (0.063) |  |
| 100-199 Inh/km2 | 0.033 | 0.034 |  | -0.065 | -0.063 |  |
|  | (0.041) | (0.041) |  | (0.063) | (0.063) |  |
| 50-99 Inh/km2 | 0.029 | 0.029 |  | 0.041 | 0.042 |  |
|  | (0.041) | (0.041) |  | (0.062) | (0.062) |  |
| <50 Inh/km2 | 0.024 | 0.025 |  | -0.033 | -0.032 |  |
|  | (0.042) | (0.042) |  | (0.065) | (0.065) |  |
| Constant | 0.042 | 0.044 | 0.293^***^ | 2.393^****^ | 2.393^****^ | 2.472^****^ |
|  | (0.103) | (0.103) | (0.097) | (0.147) | (0.147) | (0.139) |
| Observations | 799045 | 799045 | 799045 | 68155 | 68155 | 68155 |

Note: The Table reports the regression coefficients and their standard errors in parentheses; *=p<1; **=p<0.05; ***=p<0.01; ****=p<0.001; results for county and year binary indicators are omitted for brevity.
