# Supplementary material for: The effects of oral clefts on hospital use throughout the lifespan
Source: BMC Health Serv Res. 2012 Mar 9;12:58. doi: 10.1186/1472-6963-12-58 (PMC3350419; doi:10.1186/1472-6963-12-58)
Supplement: Additional file 6 — Table S6. Detailed Logistic and Poisson Regression Results for Age Group 50-59 years. [file 1472-6963-12-58-S6.DOC]

Table S6: Detailed Logistic and Poisson Regression Results for Age Group 50-59 years

|  | Logistic regression | | | Poisson regression | | |
| --- | --- | --- | --- | --- | --- | --- |
|  | Full Model | | Excluding Own SES Characteristics | Full Model | | Excluding Own SES Characteristics |
|  | *Any cleft model* | *Cleft types model* | *Any cleft model* | *Any cleft model* | *Cleft types model* | *Any cleft model* |
| Cleft Status | 0.069^*^ |  | 0.145^***^ | -0.010 |  | 0.033 |
|  | (0.042) |  | (0.043) | (0.058) |  | (0.060) |
| Cleft lip |  | -0.026 |  |  | 0.131 |  |
|  |  | (0.076) |  |  | (0.118) |  |
| Cleft lip with palate |  | 0.164^**^ |  |  | 0.016 |  |
|  |  | (0.066) |  |  | (0.081) |  |
| Cleft palate |  | 0.028 |  |  | -0.208^**^ |  |
|  |  | (0.073) |  |  | (0.098) |  |
| Male | 0.123^****^ | 0.123^****^ | -0.024^*^ | 0.097^****^ | 0.096^****^ | 0.034 |
|  | (0.016) | (0.016) | (0.014) | (0.022) | (0.022) | (0.021) |
| Age (years) | 0.016^****^ | 0.016^****^ | 0.030^****^ | 0.022^****^ | 0.022^****^ | 0.028^****^ |
|  | (0.002) | (0.002) | (0.002) | (0.004) | (0.004) | (0.004) |
| Exposure time (days) | -0.011^****^ | -0.011^****^ | -0.011^****^ | -0.003^****^ | -0.003^****^ | -0.003^****^ |
|  | (0.0002) | (0.0002) | (0.0002) | (0.0001) | (0.0001) | (0.0001) |
| Upper and post-secondary | -0.018 | -0.018 |  | 0.012 | 0.012 |  |
|  | (0.016) | (0.016) |  | (0.023) | (0.023) |  |
| Tertiary | -0.156^****^ | -0.156^****^ |  | -0.010 | -0.011 |  |
|  | (0.021) | (0.021) |  | (0.034) | (0.034) |  |
| Income quintile  20-40% | -0.166^****^ | -0.165^****^ |  | -0.050^*^ | -0.050^*^ |  |
|  | (0.019) | (0.019) |  | (0.030) | (0.030) |  |
| Income quintile  40-60% | -0.206^****^ | -0.205^****^ |  | -0.108^***^ | -0.108^***^ |  |
|  | (0.023) | (0.023) |  | (0.036) | (0.036) |  |
| Income quintile  60-80% | -0.302^****^ | -0.301^****^ |  | -0.159^****^ | -0.159^****^ |  |
|  | (0.024) | (0.024) |  | (0.041) | (0.041) |  |
| Income quintile  80-100% | -0.369^****^ | -0.368^****^ |  | -0.184^****^ | -0.185^****^ |  |
|  | (0.027) | (0.027) |  | (0.043) | (0.043) |  |
| Employed | 0.059^**^ | 0.058^**^ |  | 0.022 | 0.022 |  |
|  | (0.024) | (0.024) |  | (0.039) | (0.039) |  |
| Unemployed/other | 0.520^****^ | 0.520^****^ |  | 0.271^****^ | 0.270^****^ |  |
|  | (0.027) | (0.027) |  | (0.042) | (0.042) |  |
| Cohabiting | 0.069^***^ | 0.069^***^ |  | 0.020 | 0.020 |  |
|  | (0.026) | (0.026) |  | (0.038) | (0.038) |  |
| Single | 0.158^****^ | 0.158^****^ |  | 0.138^****^ | 0.139^****^ |  |
|  | (0.016) | (0.016) |  | (0.024) | (0.024) |  |
| 500-999 Inh/km2 | 0.013 | 0.013 |  | 0.093 | 0.092 |  |
|  | (0.044) | (0.044) |  | (0.069) | (0.069) |  |
| 200-499 Inh/km2 | 0.029 | 0.029 |  | 0.036 | 0.035 |  |
|  | (0.051) | (0.051) |  | (0.079) | (0.079) |  |
| 100-199 Inh/km2 | 0.038 | 0.038 |  | 0.048 | 0.047 |  |
|  | (0.053) | (0.053) |  | (0.080) | (0.080) |  |
| 50-99 Inh/km2 | 0.032 | 0.031 |  | 0.046 | 0.044 |  |
|  | (0.052) | (0.052) |  | (0.078) | (0.078) |  |
| <50 Inh/km2 | 0.004 | 0.004 |  | 0.014 | 0.013 |  |
|  | (0.055) | (0.055) |  | (0.082) | (0.082) |  |
| Constant | 0.812^****^ | 0.814^****^ | 0.349^**^ | 2.293^****^ | 2.292^****^ | 2.097^****^ |
|  | (0.159) | (0.159) | (0.152) | (0.220) | (0.220) | (0.214) |
| Observations | 473168 | 473168 | 473168 | 46410 | 46410 | 46410 |

Note: The Table reports the regression coefficients and their standard errors in parentheses; *=p<1; **=p<0.05; ***=p<0.01; ****=p<0.001; results for county and year binary indicators are omitted for brevity.
