# Supplementary material for: The effects of oral clefts on hospital use throughout the lifespan
Source: BMC Health Serv Res. 2012 Mar 9;12:58. doi: 10.1186/1472-6963-12-58 (PMC3350419; doi:10.1186/1472-6963-12-58)
Supplement: Additional file 7 — Table S7. Detailed Logistic and Poisson Regression Results for Age Group 60-68 years. [file 1472-6963-12-58-S7.DOC]

Table S7: Detailed Logistic and Poisson Regression Results for Age Group 60-68 years

|  | Logistic regression | | | Poisson regression | | |
| --- | --- | --- | --- | --- | --- | --- |
|  | Full Model | | Excluding Own SES Characteristics | Full Model | | Excluding Own SES Characteristics |
|  | *Any cleft model* | *Cleft types model* | *Any cleft model* | *Any cleft model* | *Cleft types model* | *Any cleft model* |
| Cleft Status | -0.024 |  | 0.022 | -0.077 |  | -0.028 |
|  | (0.081) |  | (0.081) | (0.100) |  | (0.102) |
| Cleft lip |  | -0.221 |  |  | -0.231 |  |
|  |  | (0.157) |  |  | (0.165) |  |
| Cleft lip with palate |  | 0.028 |  |  | -0.071 |  |
|  |  | (0.117) |  |  | (0.137) |  |
| Cleft palate |  | 0.071 |  |  | 0.018 |  |
|  |  | (0.156) |  |  | (0.203) |  |
| Male | 0.243^****^ | 0.244^****^ | 0.085^****^ | 0.030 | 0.031 | -0.031 |
|  | (0.025) | (0.025) | (0.024) | (0.033) | (0.033) | (0.031) |
| Age (years) | 0.015^***^ | 0.015^***^ | 0.046^****^ | 0.018^**^ | 0.018^**^ | 0.028^****^ |
|  | (0.006) | (0.006) | (0.005) | (0.008) | (0.008) | (0.008) |
| Exposure time (days) | -0.011^****^ | -0.011^****^ | -0.012^****^ | -0.003^****^ | -0.003^****^ | -0.003^****^ |
|  | (0.0004) | (0.0004) | (0.0004) | (0.0002) | (0.0002) | (0.0002) |
| Upper and post-secondary | 0.025 | 0.025 |  | 0.025 | 0.024 |  |
|  | (0.027) | (0.027) |  | (0.035) | (0.035) |  |
| Tertiary | -0.068^*^ | -0.068^*^ |  | -0.007 | -0.007 |  |
|  | (0.037) | (0.037) |  | (0.057) | (0.056) |  |
| Income quintile  20-40% | -0.179^****^ | -0.179^****^ |  | 0.034 | 0.034 |  |
|  | (0.034) | (0.034) |  | (0.041) | (0.041) |  |
| Income quintile  40-60% | -0.367^****^ | -0.367^****^ |  | -0.116^**^ | -0.117^**^ |  |
|  | (0.035) | (0.035) |  | (0.048) | (0.048) |  |
| Income quintile  60-80% | -0.288^****^ | -0.287^****^ |  | -0.092 | -0.092 |  |
|  | (0.039) | (0.039) |  | (0.057) | (0.057) |  |
| Income quintile  80-100% | -0.384^****^ | -0.384^****^ |  | -0.141^**^ | -0.142^**^ |  |
|  | (0.045) | (0.045) |  | (0.066) | (0.066) |  |
| Employed | 0.006 | 0.006 |  | -0.007 | -0.006 |  |
|  | (0.049) | (0.049) |  | (0.071) | (0.071) |  |
| Unemployed/other | 0.311^****^ | 0.311^****^ |  | 0.062 | 0.062 |  |
|  | (0.047) | (0.047) |  | (0.066) | (0.066) |  |
| Cohabiting | 0.158^***^ | 0.157^***^ |  | 0.211^***^ | 0.210^***^ |  |
|  | (0.053) | (0.053) |  | (0.070) | (0.070) |  |
| Single | 0.257^****^ | 0.256^****^ |  | 0.296^****^ | 0.295^****^ |  |
|  | (0.026) | (0.026) |  | (0.034) | (0.035) |  |
| 500-999 Inh/km2 | 0.019 | 0.019 |  | -0.186^**^ | -0.187^**^ |  |
|  | (0.074) | (0.074) |  | (0.085) | (0.085) |  |
| 200-499 Inh/km2 | 0.073 | 0.073 |  | -0.274^***^ | -0.275^***^ |  |
|  | (0.086) | (0.086) |  | (0.103) | (0.103) |  |
| 100-199 Inh/km2 | 0.076 | 0.076 |  | -0.234^**^ | -0.234^**^ |  |
|  | (0.089) | (0.089) |  | (0.107) | (0.107) |  |
| 50-99 Inh/km2 | -0.017 | -0.018 |  | -0.249^**^ | -0.250^**^ |  |
|  | (0.087) | (0.087) |  | (0.107) | (0.107) |  |
| <50 Inh/km2 | 0.007 | 0.006 |  | -0.304^***^ | -0.305^***^ |  |
|  | (0.091) | (0.091) |  | (0.109) | (0.109) |  |
| Constant | 1.074^***^ | 1.078^***^ | -0.699^*^ | 2.162^****^ | 2.159^****^ | 1.678^****^ |
|  | (0.381) | (0.381) | (0.361) | (0.503) | (0.503) | (0.471) |
| Observations | 117302 | 117302 | 117302 | 15130 | 15130 | 15130 |

Note: The Table reports the regression coefficients and their standard errors in parentheses; *=p<1; **=p<0.05; ***=p<0.01; ****=p<0.001; results for county and year binary indicators are omitted for brevity.
